# Through Hawks’ Eyes: Synthetically Reconstructing the Visual Field of a Bird in Flight

**DOI:** 10.1007/s11263-022-01733-2

**Published:** 2023-03-02

**Authors:** Sofía Miñano, Stuart Golodetz, Tommaso Cavallari, Graham K. Taylor

**Affiliations:** 1 Department of Biology, University of Oxford, 11a Mansfield Road, Oxford, OX1 3SZ UK; 2 Department of Computer Science, University of Oxford, Parks Road, Oxford, OX1 3QD UK; 3 Department of Engineering Science, University of Oxford, Oxford OX1 3PJ, UK; 4 Advanced Research Computing Centre, University College London, Gower Street, London WC1E 6BT, UK

**Keywords:** Animal vision, Visual field, Reconstruction, Bird, Flight

## Abstract

Birds of prey rely on vision to execute flight manoeuvres that are key to their survival, such as intercepting fast-moving targets or navigating through clutter. A better understanding of the role played by vision during these manoeuvres is not only relevant within the field of animal behaviour, but could also have applications for autonomous drones. In this paper, we present a novel method that uses computer vision tools to analyse the role of active vision in bird flight, and demonstrate its use to answer behavioural questions. Combining motion capture data from Harris’ hawks with a hybrid 3D model of the environment, we render RGB images, semantic maps, depth information and optic flow outputs that characterise the visual experience of the bird in flight. In contrast with previous approaches, our method allows us to consider different camera models and alternative gaze strategies for the purposes of hypothesis testing, allows us to consider visual input over the complete visual field of the bird, and is not limited by the technical specifications and performance of a head-mounted camera light enough to attach to a bird’s head in flight. We present pilot data from three sample flights: a pursuit flight, in which a hawk intercepts a moving target, and two obstacle avoidance flights. With this approach, we provide a reproducible method that facilitates the collection of large volumes of data across many individuals, opening up new avenues for data-driven models of animal behaviour.

**Supplementary Information:**

The online version contains supplementary material available at 10.1007/s11263-022-01733-2.

## Introduction

From intercepting moving targets to manoeuvring through clutter, birds use vision to coordinate their flight manoeuvres with an agility and flexibility beyond the reach of current autonomous systems. Nevertheless, the links between their vision, guidance and control are complex and poorly understood. Much more is known about the role of vision in insect flight (Taylor et al., 2008), presumably because the size and sentience of birds complicates the experimental characterisation of their visuomotor control (Altshuler & Srinivasan, 2018). Many of the previous works on avian visually guided flight followed insect studies (Baird et al., 2021; Tammero & Dickinson, 2002a, b; Altshuler & Srinivasan, 2018) and investigated the animal’s behaviour in abstract visual environments (Bhagavatula et al., 2011; Schiffner & Srinivasan, 2015; Dakin et al., 2016; Ros & Biewener, 2016), such as corridors with vertically or horizontally striped walls. This proved useful as a first step in exploring how birds use visual self-motion cues, and in isolating their effects on flight control. For example, budgerigars flying through narrow corridors regulate flight speed in response to optic flow from sliding gratings projected onto the walls (Schiffner & Srinivasan, 2015). However, these approaches oversimplify the rich visual input available to birds in their natural habitat, more so than for flying insects, since birds’ visual acuity and neural organisation is more complex (Altshuler & Srinivasan, 2018). As a result, the conclusions that can be drawn from these studies about the birds’ strategies in the wild are limited.

In this paper, we present a method for reconstructing the visual scene a bird experiences while flying through a structured environment, as a first step towards understanding how birds use visual information to guide and control their flight. Specifically, we combine high-speed motion capture data with a three-dimensional (3D) reconstruction of the laboratory environment to generate synthetic visual inputs that characterise the information likely available to the bird in flight.

We aim to support the analysis of large quantities of data across multiple individuals, using environments that may vary experimentally across trials. The synthetic inputs we can generate with our method characterise the bird’s visual experience of its own self-motion in detail over its full visual field, opening up several new avenues of research in bio-inspired computer vision and behavioural modelling.

We demonstrate the possibilities of using our method to answer mechanistic questions in behaviour with pilot data from three sample flights: one pursuit flight, in which a hawk intercepts a moving target pulled across the ground, and two obstacle avoidance flights, in which a hawk flies between two perches around a set of obstacles. In the following sections, we provide an overview of active vision in bird flight, review previous approaches to study it experimentally, and summarise the contributions of our method.

### Active Vision in Birds

In animals with well-developed visual systems, vision is largely active: through a variety of head, eye and body movements, animals can manipulate the position and orientation of their viewpoint relative to their environment (Land & Nilsson, 2012). Understanding how birds interrogate their visual environment during flight may be key to unravelling the cognitive processes coordinating their impressively fast manoeuvres.

**Fig. 4:**
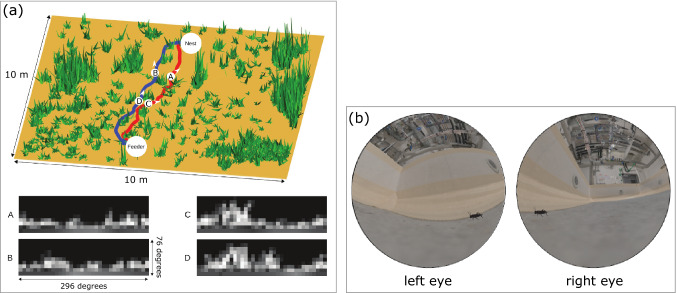
Related work. (**a**) Reconstruction of the environment around a nest of homing ants, and reconstructed views (A, B, C, D) at different instances of their recorded paths (Ardin et al., 2016). (**b**) Reconstructed view from each eye of a mouse hunting a cricket, using laser-scanned data and texture from high-resolution images (Holmgren et al., 2021). Panels (a) and (b) are reproduced from the cited works (Ardin et al., 2016; Holmgren et al., 2021) without modification under the terms of the Creative Commons Attribution License.

Birds mainly use head movements controlled by their neck motor system to look around the environment. This is because their eyes have a limited range of motion within their orbit, and the largest eye movements driven by the oculomotor system are small compared to those made by the head (Yorzinski et al., 2015; Mitkus et al., 2018; Potier et al., 2020). A small body of work has assessed how the frequency and amplitude of head movements in birds are affected by the visual environment experienced in flight. For example, in pigeons, these have been shown to vary with the structure of the landscape they are flying through (Kano et al., 2018), the structure of the clutter they are negotiating (Ros & Biewener, 2016), and the presence of another individual when flying in pairs (Taylor et al., 2019). Birds’ head movements have also been found to modulate their visual input: in turning flight, birds display a characteristic saccade-and-fixate strategy reminiscent of primate eye movements (Eckmeier et al., 2008; Ozawa, 2010; Kress et al., 2015; Ros & Biewener, 2017). This strategy supports the use of optic flow in flight control by eliminating the rotational component of the vector field during fixation, leaving only the translational component that contains the depth information (Eckmeier et al., 2008).

Although a bird’s head pose is the primary determinant of its gaze direction, eye tracking provides the most direct measure of gaze. Compared to scleral search coils (Rivers et al., 2014) or implanted magnets (Payne & Raymond, 2017), eye-tracking cameras offer the least invasive method to track eye movements. In birds, these have so far been restricted to terrestrial use cases, such as identifying where birds look when assessing mates (Yorzinski et al., 2013), watching predators (Yorzinski & Platt, 2014; Yorzinski, 2021), or inspecting the environment (Yorzinski et al., 2015). Their lack of use in flight is due to weight limitations, and the challenge of keeping the camera steady without occluding the frontal field of view.

Most bird studies therefore take head orientation as a proxy for gaze direction, which is often sufficient to identify the features to which a bird is attending. For example, work on lovebirds flying to a perch (Kress et al., 2015) investigated the alignment of the bird’s head with the edges of the perch and flight arena, whilst work on pigeons negotiating a forest of vertical poles (Lin et al., 2014) investigated the alignment of the bird’s head with the gaps between the obstacles. However, both studies analysed the problem in two dimensions, focusing only on changes in head azimuth, and reduced these extended features of the environment (selected *a priori*) to single points in the visual field. A complete understanding of the problem requires a full $360^\circ$ reconstruction of the bird’s view in flight, which is what motivates the present study.

### Related Work

Previous approaches to reconstructing what animals see of their environment have relied either on animal-borne cameras, or on reconstructing images synthetically. We review these approaches in the following sections, focusing on bird flight applications.

#### Head-Mounted Cameras

Head-mounted video cameras can sample the view of a bird as a result of its self-motion through the environment, and have been used to analyse aerial attack behaviours in hawks and falcons (Kane et al., 2015; Kane & Zamani, 2014; Ochs et al., 2016). This approach allows us to investigate a bird’s behaviour in its natural habitat, but is subject to the extreme limitations of pixel count, dynamic range and field of view of any camera small enough to mount on the head. Payload is conventionally limited to $\le 5\%$ of a bird’s body mass on welfare grounds (Fair et al., 2010), but much more stringent limits may be required to ensure natural behaviour if the load is carried on the head (Kane & Zamani, 2014). The 20 g cameras that have been used previously (Kane et al., 2015; Kane & Zamani, 2014) are twice the weight of many small birds, and therefore only suitable for very large species such as raptors. Even so, it is currently not possible for a small camera to cover a bird’s full field of view at an appropriate optical or sampling resolution. For example, the vertical field of view ($31^\circ$) of the head-mounted camera used to study goshawks and falcons (Kane et al., 2015; Kane & Zamani, 2014) wouldn’t cover the vertical extent of the binocular overlap ($100^\circ$) of the birds in this work, namely Harris’ hawks (Potier et al., 2016). Furthermore, the possibilities for analysing head-mounted video data are also impacted by the cameras’ low frame rates (30 Hz was used in Kane et al. , 2015, and in Kane and Zamani , 2014), and the motion blur associated with low shutter speeds and rolling shutters (Kane & Zamani, 2014). Finally, although head-mounted cameras can be held reasonably fixed relative to a raptor’s head using a tightly fitted hood (Kane et al., 2015), fitting a hood may not be a possibility in untrained or smaller birds. Generally, head-mounted cameras will be prone to some degree of wobble unless surgically attached to the head (Lev-Ari & Gutfreund, 2018; Hazan et al., 2015), which is an undesirable intervention. Head-mounted video cameras therefore have less utility for studying visually guided flight in birds than might first be imagined.

#### Synthetic Reconstruction

An animal’s visual input can be recreated synthetically using bio-inspired hardware, for example with custom-designed cameras (Stuerzl et al., 2010) or event-based cameras (Zhu et al., 2021; Gallego et al., 2022). It can also be done via software, using rendering methods (Holmgren et al., 2021). Rendering is particularly attractive because it offers complete control of the detail presented over the visual field (Holmgren et al., 2021; Neumann, 2002), and it is well suited to scientific inference because of the possibility of defining alternative views (Eckmeier et al., 2013; Ravi et al., 2022; Miñano & Taylor, 2021; Bian et al., 2021). For example, in a series of works studying the effectiveness of movement-based signalling in lizards, renderings were used to investigate the effect of different lighting and wind conditions (Bian et al., 2018, 2019, 2021).

State-of-the-art research has proven it possible to render novel views from only a set of camera views and poses (Tancik et al., 2022a; Mildenhall et al., 2020). However, while user-friendly approaches are emerging for the use of these cutting-edge techniques among non-experts (Tancik et al., 2022b), most current rendering applications still require an explicit 3D model of the environment. There are some challenges involved in realistically modelling a natural-looking 3D environment. Standard modelling approaches such as simultaneous localization and mapping (SLAM) suffer from accumulating noise and drift when covering large areas (Schonberger & Frahm, 2016), and automating the post-processing of the resulting meshes may not be straight forward (Risse et al., 2018; Stürzl et al., 2015). Nevertheless, the quality of the dense maps that can now be captured with consumer-level handheld devices has improved greatly in the past few years, achieving results comparable to more expensive laser-scanning methods even in complex forest environments (Tatsumi et al., 2022; Gollob et al., 2021).

Most synthetic reconstructions of an animal’s visual scene to date have focused on insects. Insects are generally simpler to model than vertebrates, due to their lower sampling resolution, and the fact that their eyes are rigidly fixed to their heads. A few studies have investigated ant navigation using fully synthetic models of the natural environment (Ardin et al. , 2015; Ardin et al. , 2016; see Fig. 1a), and panoramic images of the ants’ habitat (Zeil et al., 2014). The role of optic flow in bee flight has been analysed using a basic geometric reconstruction of the laboratory environment (Ravi et al., 2019, 2022), whereas the homing flight of bees and wasps has been studied using detailed 3D models of their natural environment (Stürzl et al., 2015; Stuerzl et al., 2016; Schulte et al., 2019). The latter used models obtained using laser scanners, structure-from-motion (SfM), and photographic reconstruction techniques.

Although vertebrates generally have more complex visual systems than insects, the same general approaches have been extended to study their visually guided behaviours. One recent study analysed prey pursuit in mice by tracking the animal’s head and eye movements, and combining them with a high-resolution 3D laser scan of the lab environment (Holmgren et al. , 2021; see Fig. 1b). An earlier work in zebra finches reconstructed a simplified view of a bird in a single turning flight (Eckmeier et al., 2013), using a basic geometric model of the flight arena and the bill’s orientation as a proxy for gaze direction. In both of these studies, the environments mapped were $< 1 \hbox {m}^{3}$, but there is currently growing interest in reconstructing an animal’s experience of its environment at much larger scales, relevant to ecology and conservation (Tuia et al., 2022). This interest has led to demonstrations of animal-borne 3D mapping sensors (McClune, 2018), and a mobile-camera method for embedding an animal’s track in an aerial view of its environment (Haalck et al., 2020).

Fully synthetic renderings also lend themselves to being used in a virtual reality (VR) environment. This approach has been applied to tethered and freely moving animals, mostly insects (Kern et al., 2005; Taylor et al., 2008; Windsor & Taylor, 2017), but also more recently small vertebrates. Examples include restrained birds (Eckmeier et al., 2013) and freely moving mice and zebra fish (Stowers et al., 2017; Naik et al., 2020). Such environments are currently limited to volumes of approximately 1 m$^3$, so have yet to find use for larger animals making larger-scale movements.

**Fig. 5:**
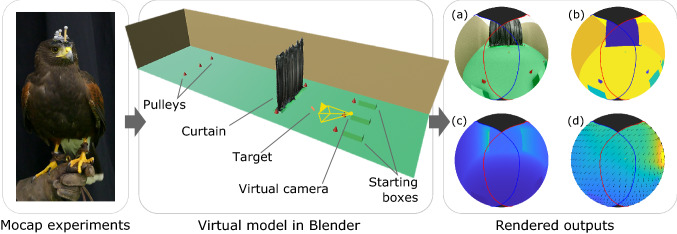
Summary of method for reconstructing the visual information contained within the visual field of a bird in flight. The head movements of birds executing flight manoeuvres are recorded in a large motion capture lab (left panel). The lab environment is modelled in Blender, using geometric primitives and a dense 3D map to model objects with a more complex geometry (centre panel). The measured head pose is then used to define a virtual camera that is representative of the bird’s visual field (right panel). With this approach, we can generate detailed information describing the visual scene that the bird experiences in flight, including: (**a**) RGB renderings; (**b**) semantic maps; (**c**) depth maps; and/or (**d**) optic flow. In the virtual model of the lab (centre panel), the pulleys and target are displayed at twice their actual size for clarity. The spheres representing the bird’s visual field in the right panel show the animal’s view as it flies through the lab. The retinal margins of the left eye (blue line) and right eye (red line) are shown for reference, as well as the blind sector above the bird’s head (black fill). Note that because the spheres are represented using an orthographic projection, not all the visual field of the bird is visible

### Contribution

We describe a method to render the visual scene experienced by a bird in flight, combining high-speed motion capture with 3D modelling of the laboratory environment. The data rendered from the bird’s perspective includes a rich set of outputs: RGB, semantic, depth and optic flow maps over the complete visual field of the bird (see Fig. 2). Although analogous data have been generated for insects, these were produced at much lower spatial resolution (Ravi et al., 2022, 2019; Schulte et al., 2019; Stuerzl et al., 2016; Stürzl et al., 2015). Additionally, none of these previous works involved a unique method to produce the full set of outputs we consider here. In birds, a similar RGB reconstruction has been previously generated for a single flight of a zebra finch (Eckmeier et al., 2013). However, this is the first time, to our knowledge, that such detailed data have been produced for large birds in flight, capturing their full visual fields and the full 6 degrees-of-freedom of their heads’ motion.

Compared to previous approaches to characterise the visual input of a bird in flight, our method has significant advantages:

- It considers the complete visual field of the bird. As a result, posterior analyses are not limited by the available field of view of a head-mounted camera (Ochs et al., 2016; Kane et al., 2015; Kane & Zamani, 2014), or to local features of the scene falling in the direction of the bird’s gaze (Eckmeier et al., 2008; Yorzinski et al., 2013; Yorzinski & Platt, 2014; Yorzinski et al., 2015; Kress et al., 2015; Yorzinski, 2021).
- It is not limited by other technical specifications of a camera that is practical to attach to a bird’s head for use in flight.
- It is minimally invasive, which is preferred both on welfare grounds, and to preserve the animal’s natural behaviour as much as possible. The total weight carried by the bird is 3 g, much lower than the 20 g of a typical head-mounted camera (Kane et al., 2015; Kane & Zamani, 2014).
- It allows us to consider different camera models and gaze strategies for the purposes of hypothesis testing.
- The method is designed to support the collection of large amounts of data across different individuals, in environments that may vary experimentally across trials.
- It is able to take advantage of a 3D modelling approach that can be adapted to the required level of detail and realism.
- It combines several computer vision techniques (high-speed motion capture, 3D mapping, rendering and coordinate system registration) in a novel way enabling application in the field to investigate animal behaviour.

We demonstrate how our method can provide a unique insight into the hawks’ visually guided behaviour with simple behavioural analyses on three sample flights. However, the method would be useful too in more sophisticated and novel approaches to animal behaviour, for example to develop data-driven models of animal visuomotor control (Zhang et al., 2018; Merel et al., 2020), to provide realistic stimuli relevant for neural recording experiments in VR setups (Eckmeier et al., 2013), or as a first step towards fully synthetic models of an animal’s behaviour (Neumann, 2002). To support further work in these directions, we will provide the code for the rendering pipeline shortly after publication at https://github.com/sfmig/hawk-eyes.

**Fig. 6:**
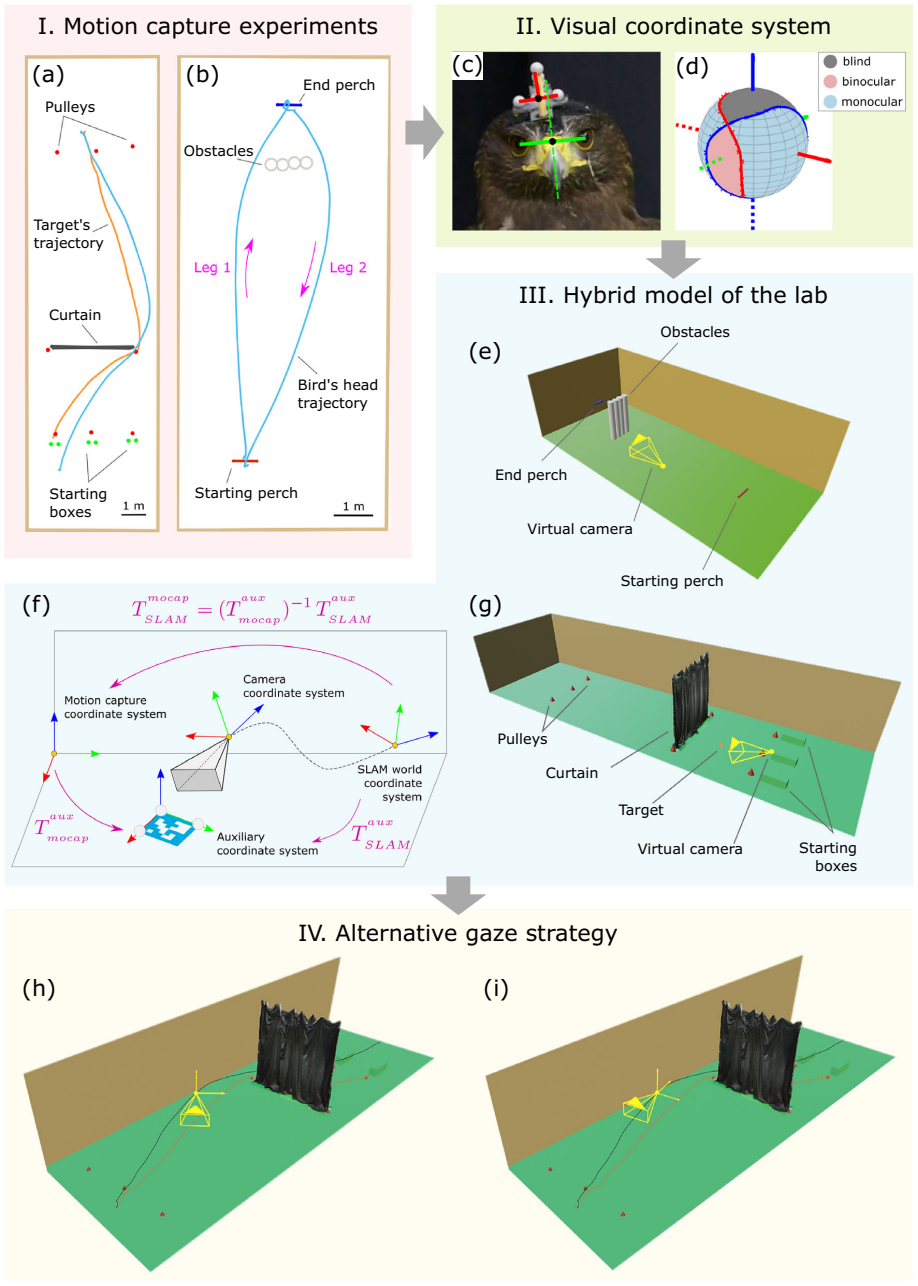
Key features of the method. Panel I: we carried out motion capture experiments with Harris’ hawks, in which we tracked their head movements while executing pursuit (**a**) and obstacle avoidance manoeuvres (**b**). We used additional markers to locate the main elements of the scene. The pursuit flight takes 2.5 s and the obstacle flights around 2 s each. Note that the two obstacle avoidance flights correspond to the two legs of the same trial (magenta arrows and text). Panel II: we used the motion capture data to estimate the transform from a headpack coordinate system (**c**, shown schematically in red) to a coordinate system representative of the bird’s visual field (**c**, shown schematically in green). We used data available in the literature to estimate the monocular, binocular and blind regions on the bird’s visual coordinate system (**d**). Panel III: we propose to model the lab environment with a hybrid approach, which uses a combination of geometric primitives for the simple geometries in the scene, and dense 3D meshes for the more complex ones. To facilitate the integration of the captured dense 3D maps in the motion capture coordinate system, we transform them at the point of acquisition using an ArUco fiducial marker (**f**). The transforms between coordinate systems are shown (magenta text), where $T_{A}^{B}$ represents the transform from *A* to *B*. We demonstrate this hybrid approach for the pursuit flight (**g**), modelling the curtain with a dense 3D map. For the obstacle avoidance flights, we used geometric primitives only (**e**). Panel IV: our method allows us to define alternative gaze strategies for the purpose of hypothesis testing. We demonstrate this by defining two scenarios for each of the flights. In the first scenario (**h**), the virtual camera (yellow) tracks the visual coordinate system, which we expect to be representative of the pose of the bird’s visual field. In the second one (**i**), the virtual camera tracks the trajectory coordinate system, which represents a horizon-level camera whose optical axis is tangent to the bird’s head trajectory (black line). This is shown for the pursuit flight in the figure, along with the target’s trajectory (orange line). The virtual camera is represented schematically as a pyramid, but note a $360^\circ$ virtual camera was used for all renderings

## Methods

In this section, we describe the key details of the motion capture experiments and of their synthetic reconstruction in a computational environment.

### Motion Capture Experiments

We recorded Harris’ hawks (*Parabuteo unicinctus*) flying in a large ($20\times 6\times 3.3$ m) motion capture lab, using 22 Vicon Vantage V16 motion capture cameras sampling at 200 Hz (Vicon Motion Systems Ltd., Oxford, UK). Here we present results for $n=3$ sample flights from two different birds, executing pursuit and obstacle avoidance manoeuvres. These flights are part of a larger dataset of over $>100$ trials across 5 individuals. We use this small subset of flights to describe and illustrate the method, and a complete description of the full set of experiments will be provided separately elsewhere.

#### Bird Flights

For the pursuit flight, the bird (*Toothless*) chased a cylindrical artificial target with food reward (length: 0.15 m; diameter: 0.025 m) that was dragged in an unpredictable direction around a series of pulleys at an average speed of 5.6 m s$^{-1}$. To further challenge the bird’s manoeuvring, we hung a black curtain across the room from floor to ceiling, leaving a gap of approximately one wingspan ($1.0-1.1$ m) to either side, through which the bird and target passed (Fig. 3a).

For the obstacle avoidance flights, the bird (*Drogon*) flew between two perches set 9 m apart, and around a set of four cylindrical styrofoam pillars (height: 2 m; diamater: 0.3 m) placed 1.5 m in front of one the perches (Fig. 3b). Note that the two flights correspond to the two legs of the same trial (see magenta arrows in Fig. 3b). Results from the two obstacle avoidance flights have also been presented in the preprint by Miñano and Taylor (2021), using a slightly different analysis approach. The walls of the laboratory environment were hung with camouflage netting, and other reconstruction cases that we trialled included placing small trees within this environment. Further details on the experimental setup and the birds can be found in Appendix A.

#### Motion Capture Data

We tracked the bird’s head, using a custom ‘headpack’ comprising a rigid arrangement of four or five 6.4 mm diameter spherical retroreflective markers that we fixed to a Velcro patch glued to the bird’s head (see Appendix 1). We tracked the target using three 6.4 mm diameter markers, and attached further 6.4 or 14 mm diameter markers to the main static elements of the scene. We used Nexus v2.8.0 software (Vicon Motion Systems Ltd., Oxford, UK) to extract the 3D positions of all the unlabelled retroreflective markers. For the pursuit flight, we labelled the headpack markers manually within Nexus, using its semi-automatic labelling functionality. For the obstacle avoidance flights, we labelled the headpack markers automatically using custom scripts written in MATLAB R2020b (The Mathworks Inc., Natick, MA). In both cases we used custom MATLAB scripts to label stationary obstacle markers, to compute and interpolate the pose of the headpack and target, and to handle missing marker data. Further detail on these post-processing steps is presented in B.

#### Calibration of the Bird’s Visual Coordinate System

The headpack was arbitrarily placed on the bird’s head before the experiments. As a result, a coordinate system defined relative to the headpack markers is not necessarily aligned with the principal axes of the bird’s visual field (Fig. 3c). To estimate the bird’s visual coordinate system, we make use of three assumptions (Miñano & Taylor, 2021). First, we assume that the bird’s gaze movements across the environment are largely executed via head movements, and that the eyes’ movement relative to the head is small (Kano et al., 2018; Brighton et al., 2017; Ros & Biewener, 2017; Kress et al., 2015; Kane & Zamani, 2014; Eckmeier et al., 2008). Second, we assume that the bird’s gaze direction is known from first principles during calibration and we identify it with the forward direction of the head. In the pursuit case, we assume that the bird looks at food presented to it by the falconer; in the obstacle avoidance case, we assume that the bird looks at the perch centre upon landing (Potier et al., 2016; Kress et al., 2015). Third, we assume that the bird holds its eyes level during the calibration. This eye-levelling behaviour has been reported repeatedly in the bird flight literature (Brighton & Taylor, 2019; Ros & Biewener, 2017; Warrick et al., 2002), and is confirmed by our reference videos too. Further detail on the visual coordinate system calibration is included in C.

We additionally determined the monocular, binocular and blind areas of the visual field of a Harris’ hawk in a sphere centred at the origin of the estimated visual coordinate system (Fig. 3d). To do this, we digitised and interpolated the data available in the literature (Potier et al. , 2016, Figures 5C and 6), and assumed the gaze direction of our visual coordinate system corresponded to the direction of maximum binocular overlap. The same method, further described in Appendix C.4, could be used in other animal species, given the data that is typically published to describe the visual field of an animal.

#### Ethics Statement

This work has received approval from the Animal Welfare and Ethical Review Board of the Department of Zoology, University of Oxford, in accordance with University policy on the use of protected animals for scientific research, permit no. APA/1/5/ZOO/NASPA, and is considered not to pose any significant risk of causing pain, suffering, damage or lasting harm to the animals. No adverse effects were noted during the trials.

### Computational Model in Blender

We defined a computational model of the motion capture experiments in Blender (Blender Online Community, 2021), a 3D modelling software package with a rendering engine. This involved: (i) defining a virtual camera, representative of the bird’s perspective in flight, and (ii) defining a 3D model of the lab geometry during the experiments. The code to generate the model of the lab environment and define the corresponding virtual camera in Blender will be made available at https://github.com/sfmig/hawk-eyes.

#### Virtual Camera

We modelled the scene viewed by the bird using a 360$^\circ$ virtual camera whose translation and rotation per frame matched those of the estimated visual coordinate system (Fig. 3h). Since any vergence movements of the eyes are unknown, we modelled the bird’s binocular visual system as a monocular camera. We selected a resolution of 5 pixels per degree latitude and longitude. This results in all pixels within the bird’s visible region (monocular plus binocular) having a length and width $\sim 10\times$ the minimum resolution angle of the bird at its fovea (Potier et al., 2016). Note that we consider a uniform resolution across the camera’s full visual field, but this is not the case for the hawks: their visual acuity varies across their retinas and is highest at the foveae (Mitkus et al., 2018; Potier et al., 2016). For each flight, we rendered the view from this virtual camera using the Cycles rendering engine, and produced RGB, depth, semantic and optic flow data per pixel, for each motion capture frame (sampled at 200 Hz).

To test the effect of the bird’s head movements, we defined an alternative gaze strategy. This is represented by a horizon-levelled virtual camera whose optical axis is always tangent to the bird’s head trajectory (see Fig. 3i). Specifically, the virtual camera follows a trajectory coordinate system, whose y-axis is defined parallel to the bird’s head velocity vector, whose x-axis is parallel to the floor plane, and whose origin is that of the visual coordinate system (see Appendix C.5). For each flight, we rendered the view from this virtual camera as well.

#### Hybrid 3D Model of the Lab

We model the lab environment using a hybrid approach, which uses a combination of geometric primitives for the simple geometries in the scene, and dense 3D meshes for the most complicated ones (Fig. 3, panel III). The dense 3D meshes are captured with a mobile device, and expressed at acquisition time in the same coordinate system as the motion capture trajectories. This way we minimise the modelling and postprocessing effort, while producing realistic representations of the environment.

We demonstrate the use of a hybrid model of the lab for the pursuit flight, modelling the curtain using a dense 3D mesh, and the rest of the objects in the scene as geometric primitives (Fig. 3g). In the obstacle avoidance flights, we used geometric primitives only (Fig. 3e).

#### Dense 3D Map

To capture a dense 3D map of the curtain in the pursuit flight, we used the open-source *SemanticPaint* framework (Golodetz et al., 2015, 2018), which is built on top of InfiniTAM v3 (Prisacariu et al., 2017). We used the ASUS ZenFone AR smartphone as a mobile mapping sensor, and to perform visual-inertial odometry (ZenFone ZS571KL, ASUS, Taipei, Taiwan). To facilitate the integration of the dense map in the virtual model of the lab, we registered it to the motion capture coordinate system, using ArUco fiducial markers (Romero-Ramirez et al., 2018; Garrido-Jurado et al., 2014). The voxel size was set to 10 mm and the truncation distance to 40 mm ($4\times$ the voxel size).

Figure 3f summarises the coordinate transformations applied to a captured dense 3D map to express it in the motion capture coordinate system. The 3D mesh is initially expressed in the SLAM world coordinate system, which is defined by default as the first camera pose. To compute the required transform from the SLAM world coordinate system to the motion capture coordinate system, we used an ArUco calibration plate. This consisted of an ArUco fiducial marker of size $28.8 \times 28.8$ cm fixed to an acrylic plastic sheet with three retroreflective markers (10 mm diameter) on three of its corners. When brought into camera view, the coordinates of the ArUco marker’s corners are computed in the SLAM world coordinate system. Since we also placed retroreflective markers on these corners, their coordinates in the motion capture coordinate system are also known. By defining an auxiliary coordinate system with these three points, we can compute the transform from the SLAM world coordinate system to the motion capture one.

We used the open-source software MeshLab (Cignoni et al., 2008) to crop the mesh, remove duplicate vertices, and remove isolated pieces. We found that the floor plane of the mesh was slightly deviated from the motion capture system’s xy-plane ($2.4^\circ$, see Appendix D.3), likely due to drift. We used MATLAB’s Point Cloud Processing functions to fit a plane to the floor of the mesh (mean error $=0.038$ m), and transform it to the motion capture’s xy-plane. The transformed mesh deviated on average by 0.093 m from the reference markers placed on the curtain’s edges, as they were registered during the pursuit trial, and by 0.089 m from their position recorded just before capturing the mesh. Further details on the postprocessing of the mesh and the deviation metrics are included in Appendix D.3.

#### Geometric Primitives

We modelled the floor, ceiling and walls of the motion capture lab as planes. The floor plane was computed during the calibration of the motion capture system, and we determined the walls and ceiling planes from the motion capture cameras’ positions and orientations (see Appendix B.4).

In the pursuit flight, we modelled the pulleys as cones and the boxes covering the target’s initial position as cuboids (Fig. 3g). We modelled the target as a cylinder of 15 cm length and 2.54 cm diameter. In the obstacle avoidance flights, we modelled the obstacles as vertical cylinders, and the perches as horizontal cylinders, thereby reducing each A-frame perch to the top rung on which the bird landed (Fig. 3e). The position, orientation and size of all these geometric elements was determined from the retroreflective markers attached to the corresponding objects, and from measurements of the dimensions of the real objects. We placed reference markers on the curtain’s edges and on the wall netting at the curtain gap, but only used them to measure deviation from our modelled geometry (see Appendices A.3 and D.3). Further details on the definition of the geometric primitives are included in Appendix D.

Textures can also easily be added to make the synthetic scene as photo-realistic as required. As an example, we include an RGB rendering of the pursuit flight in which the walls of the lab model are textured, replicating the camouflage netting that was hung in the lab to prevent the birds from perching (see Online Resources 1 and 2).

**Fig. 7:**
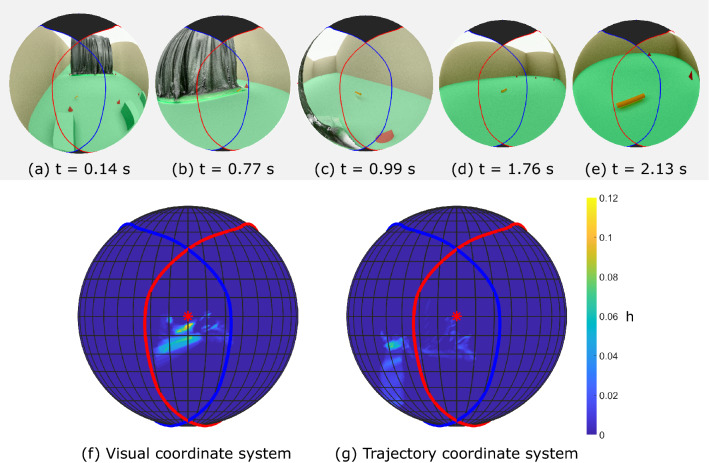
Snapshots of the rendered pursuit flight and semantic heatmaps of the target. The snapshots of the RGB rendering (**a**–**e**) represent the bird’s view as it flies through the lab in the pursuit flight. The caudal blind area is shown (black fill). The full flight takes 2.5 s. The heatmaps (**f** and **g**) show the value of *h* (Eq. 1), representing the frequency with which the target appears at each pixel in the visual field throughout a flight, normalised by the relative area of the solid angle that each pixel subtends. Results are shown for a virtual camera following the visual coordinate system (**f**), and the trajectory coordinate system (**g**). The camera axis (red star) corresponds to the estimated direction of the bird’s gaze $\vec {v}_{gaze}$ in the visual coordinate system, and to the direction of the head’s velocity vector in the trajectory coordinate system. The retinal margins for the left eye (blue line) and right eye (red line) are shown for reference. Note that because the visual field spheres are represented using an orthographic projection, not all of the bird’s visual field is shown (see Fig. 26)

## Results

For the three sample flights (one showing a pursuit manoeuvre and two recording obstacle avoidance manoeuvres), we rendered the view from: (i) a virtual camera aligned with the visual coordinate system, representing the bird’s visual field inclusive of all head movements; and (ii) a virtual camera following the trajectory coordinate system, representing the bird’s visual field exclusive of the animal’s rotational head movements. The rendered outputs per frame (RGB, depth, semantic and optic flow data) are included as supplementary videos (Online Resources 1–15, see Table 1). Further details on how these videos were produced are included in the Supplementary information and in Appendix 1.

We used the RGB and semantic data per frame to inspect the birds’ gaze strategy in the pursuit and obstacle avoidance manoeuvres, as a demonstration of how this approach can offer new insights into the bird’s behaviour. We followed a similar approach to the preliminary analysis presented by Miñano and Taylor (2021).

**Fig. 8:**
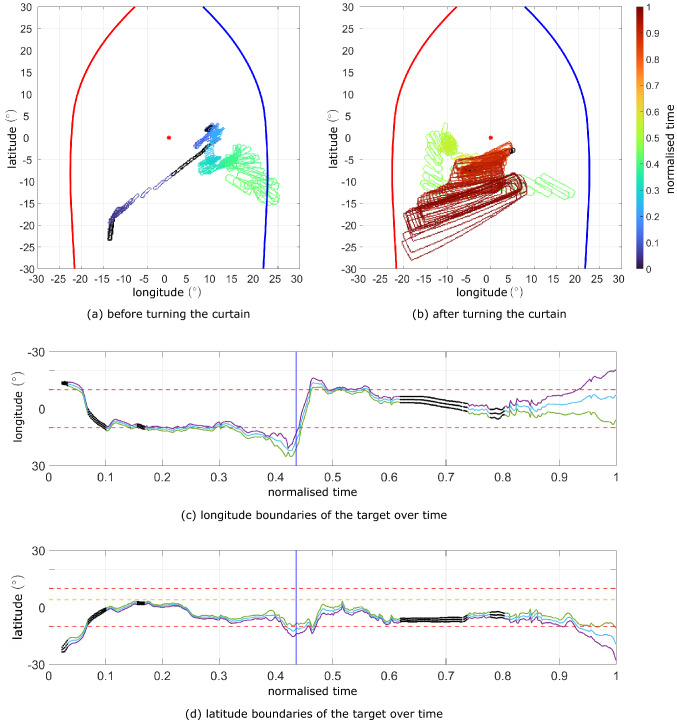
Trajectory of the target in the bird’s visual field. The edge contour of the target is represented for each frame of the pursuit flight in a cropped equirectangular projection of the area around the estimated gaze direction $\vec {v}_{gaze}$ (red star), for the frames before (**a**) and after (**b**) turning around the curtain. The colormap indicates normalised time through the flight. The extension of the target in longitude and latitude over time is shown in (**c**) and (**d**) respectively, with the maximum (purple), minimum (green), and mean (blue) values represented per frame. For those frames in which the bird’s head transform was interpolated, these values are shown in black. The blue vertical line in (**c**) and (**d**) indicates the frame that defines the data split before (**a**) and after (**b**) turning the curtain. Reference lines are shown in (**c**) and (**d**) at $\pm 10^\circ$ (red dashed lines) and at $4^\circ$ latitude in (**d**) (yellow dashed line). The normalised time is 0 at the takeoff frame, identified when the bird dips its head just before the takeoff jump; note that it takes some frames for the target to become visible, but the linear motor pulling it was already triggered at this point. The normalised time is 1 at interception, when the bird and the target reach a local minimum of distance in the terminal phase of interception. The data shown cover 2.145 s

**Fig. 9:**
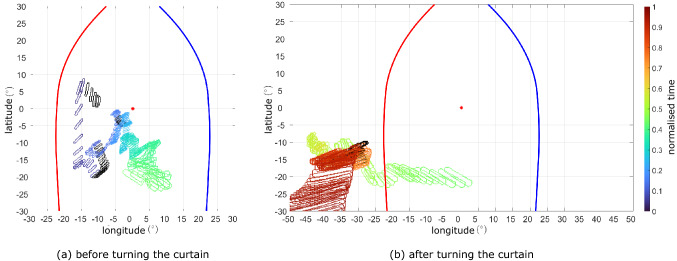
Trajectory of the target in the trajectory coordinate system. The edge contour of the target is represented for each frame of the pursuit flight in a cropped equirectangular projection of the area around the camera axis (red star) in the trajectory coordinate system. The virtual camera’s axis corresponds to the direction of the head’s velocity vector. The extent of the retinal margins of the bird’s right eye (red) and left eye (blue) is shown relative to the virtual camera’s axis for reference, although they are not expected to be positioned correctly or consistently in this coordinate system. The data is split following the same criteria as in Fig. 5, separating the frames before (**a**) and after (**b**) turning around the curtain. The colormap indicates normalised time through the trial. Note that in the first part of the flight the target shows prominent pitch oscillations likely reflecting the reaction to the wingbeat. In the second part of the flight, the target is not constrained to the equivalent of the binocular area, showing that the velocity vector of the head trajectory is not aligned with the target

### Pursuit Flight

The RGB renderings in the visual coordinate system show that the target remains within the bird’s area of binocular overlap for almost the entire duration of the pursuit (see Fig. 4a–e, and Online Resources 1 and 2). In contrast, when the camera tracks the trajectory coordinate system the target is not held steady or centered, and the RGB renderings display pronounced pitch oscillations. This comparison confirms that the bird uses its rotational head movements to stabilize its gaze, and to keep the target reasonably well centered within its visual field.

To analyse these behaviour quantitatively, we use the semantic data. Figure 4f shows the frequency with which the target appears at each point in the visual field during the flight. For each pixel, the figure displays the metric:

1 $$\begin{aligned} h_i = \frac{n_i}{N A_i}, \end{aligned}$$

where $n_i$ denotes the number of frames over which the *i*th pixel saw the target, *N* denotes the total number of frames analysed, and $A_i$ denotes the solid angle subtended by the *i*th pixel, normalised by the maximum solid angle that any pixel subtends. Note that different pixels subtend different solid angles, due to the semantic output being an equirectangular projection of a sphere. We only consider pixels within the visible areas of the bird’s visual field, and exclude from the analysis any frames in which the head transform was interpolated, and any frames after interception. The results show that the target is held within $\pm 10^\circ$ longitude and from $-10^\circ$ to $4^\circ$ latitude in the visual coordinate system for most of the flight (Fig. 4f). This is in sharp contrast with the results in the trajectory coordinate system, in which the target is not confined to this central area at all (Fig. 4g). However, a limitation of these results is that they are affected by the apparent size of the target, and refer to data aggregated across the whole of the flight. How does the target’s position in the visual field vary along the flight?

Figure 5 plots the evolution of the target’s contour in the visual coordinate system. The visual field is cropped close to the binocular area and shown in equirectangular projection. The sections of flight before and after the curtain are plotted separately for clarity (Fig. 5a and b respectively). In the first section of the flight, the target begins drifting across the visual field, but then seems to be stabilised at approximately $10^\circ$ longitude (Fig. 5a). Target tracking seems to be lost temporarily as the bird turns around the curtain (green contours in Fig. 5a), but is quickly recovered with the target now stabilised at $-10^\circ$ longitude (Fig. 5b). Towards the end of the flight, the target gradually becomes centred in the visual field, looming until interception. The same evolution can be seen by inspecting the longitudinal position of the boundaries and midpoint of the target through time (Fig. 5c). The target’s boundaries also remain between $-10^\circ$ and $4^\circ$ latitude for most of the flight (Fig. 5d).

For comparison, we computed the equivalent path of the target’s contour as seen from the trajectory coordinate system (Fig. 6). In the first part of the flight, the target shows considerably more oscillations in the vertical direction, likely due to the wingbeat motion (Fig. 6a). Just before turning around the curtain, the target appears to be aligned longitudinally with the bird’s head velocity vector. In the second part of the flight (Fig. 6b), the target is clearly not aligned with the head’s velocity vector, drifting out of the central area of the trajectory coordinate system (Fig. 6b). The comparison of Figs.  5b and 6b reflects how the estimated gaze direction, which approximates the forward direction of the bird’s head, diverges from the bird’s velocity vector in the final phase of interception.

**Fig. 10:**
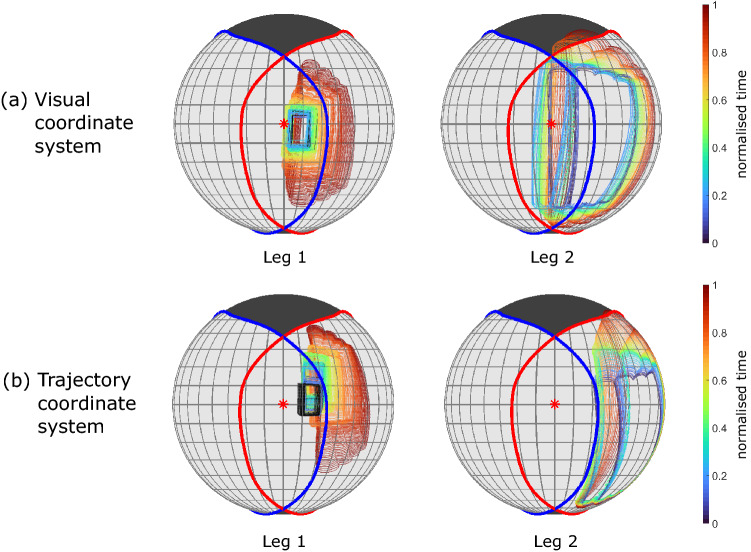
Trajectory of the obstacles in the visual field sphere. The edge contour of the set of obstacles is represented in the virtual camera’s visual field for each frame (coloured semi-transparent) of both obstacle avoidance flights, from the point of takeoff to the point at which the perch is fully visible (i.e. until the second blue dashed vertical line in Fig. 8). The colormap indicates normalised time through each flight. For the frames in which the camera’s transform was interpolated (i.e. because not enough markers were reconstructed), the contour of the obstacles is shown in black. The retinal margins of Harris’ hawks are shown for the left eye (blue line) and right eye (red line), and the virtual camera’s axis is shown for reference (red star). Note that the virtual camera’s axis corresponds to the estimated gaze direction $\vec {v}_{gaze}$ in the visual coordinate system (a), and to the direction of the head’s velocity vector in the trajectory coordinate system (b) In the visual coordinate system, the leftmost edge of the set of obstacles stays largely aligned with the estimated sagittal plane (i.e., the symmetry plane of the head) in both flights. In contrast, in the trajectory coordinate system, the obstacles are not stabilised in the vertical direction and they are not aligned with the head velocity vector either. The visible area in the bird’s visual field extends beyond what is shown in this orthographic projection (Color figure online)

### Obstacle Avoidance Flights

In both obstacle avoidance flights, the RGB renderings in the visual coordinate system show the obstacles centred in the bird’s visual field (see Online Resources 8 and 9 for the flight corresponding to leg 1 of the trial, and Online Resources 12 and 13 for the flight corresponding to leg 2). This is not the case when inspecting the RGB renderings in the trajectory coordinate system. In them the obstacles are not centred, and oscillations are clearly visible (see Online Resources 10 and 11 for leg 1 of the trial, and Online Resources 14 and 15 for leg 2). This again confirms that the bird actively stabilises its visual field against the pitch oscillations associated with its wingbeat, and also directs its gaze so as to keep the obstacles broadly centered. In this case, however, close inspection of the semantic data reveals a more subtle interpretation of how the bird is directing its gaze.

We display the evolution of the obstacles’ contour in Fig. 7. We use an orthographic projection, rather than the equirectangular one we used for the pursuit flight, to reduce the distortion, since the obstacles occupy a much larger portion of the field of view than the target. The obstacles’ contour is represented from the takeoff frame until the frame at which the landing perch is visible without occlusion. The data show that the nearside edge of the obstacles as seen by the bird remains aligned longitudinally with the centre of the visual coordinate system in both flights (Fig. 7, top row). This alignment does not appear for the data rendered in the trajectory coordinate system (Fig. 7, bottom row), which reinforces the role of the bird’s head movements in fixating the obstacles’ nearside edge.

We can inspect the bird’s attention on the obstacles and the landing perch by combining the semantic data from these two elements. Figure 8 plots how the longitudinal positions of the midpoint and edges of the obstacles and the landing perch evolve through time. For the obstacle avoidance flight comprising the first leg of the trial, the nearside edge of the obstacles appears to remain approximately aligned with the centre of the visual field until the point at which the landing perch first becomes fully visible (Fig. 8a). Beyond this point, the midpoint of the landing perch becomes the object most closely aligned with the centre of the visual field. For the obstacle avoidance flight comprising the second leg of the trial, the nearside edge of the obstacles is again aligned closely with the bird’s estimated gaze direction until the point at which the landing perch becomes visible. Then, the bird appears to make a head saccade such that its new gaze direction aligns with the nearside edge of the landing perch. This remains the case for approximately the next 0.8 s (Fig. 8b), after which the bird seems to make another head saccade, to realign its head forward direction with the midpoint of the perch. Both head saccades can be seen in the corresponding RGB rendered videos (Online Resources 12 and 13).

**Fig. 11:**
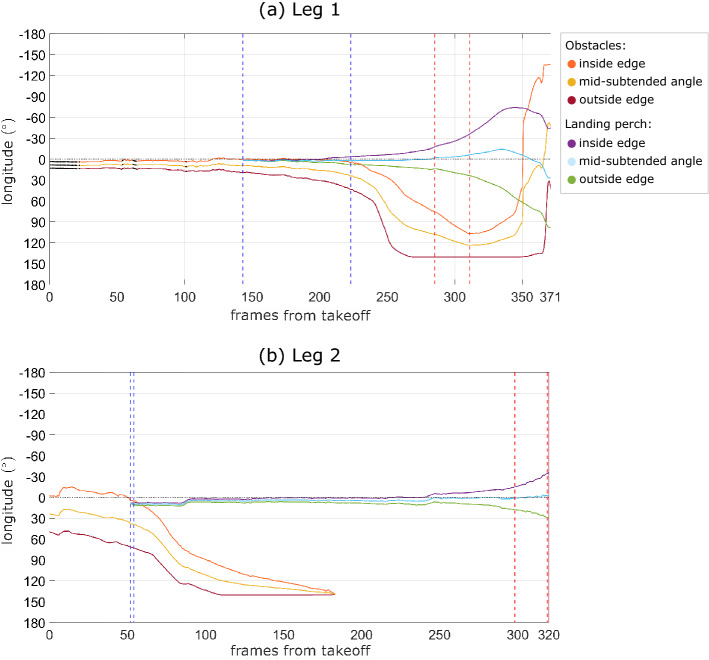
Longitudinal extension of the set of obstacles and landing perch for the obstacle avoidance flights. The evolution through time of the longitudinal extension of the obstacles and landing perch is represented for the two obstacle avoidance flights, corresponding to leg 1 (a) and leg 2 (b) of the trial. The inside and outside edges of the obstacles and landing perch are labelled relative to the bird’s turn, together with the midpoint of the angle subtended by the obstacles and landing perch. Black markers denote frames in which the bird’s head transform was interpolated. The range of frames when the landing perch is partially occluded by the obstacles is marked between two vertical dashed lines. Note that because objects curve when they are close to the poles of the spherical virtual camera, it may be that the landing perch is fully visible but that its longitudinal extension overlaps with that of the obstacles (e.g. at around 225 frames from takeoff; see Online Resources 8 and 9). The first and last frames used to estimate the bird’s gaze direction $\vec {v}_{gaze}$ are marked with vertical red dashed lines. In leg 1 of the flight (a), the obstacles appear within the central part of the visible field after 350 frames, but in reality they would have been occluded by the bird’s body (see Online Resource 8). In leg 2 (b), we can visually identify two potential head saccades: one at 87 frames after takeoff seems to align the estimated gaze direction with the left edge of the landing perch, as seen by the bird; the other at 242 frames after takeoff seems to align the estimated gaze direction with the centre of the perch (see Online Resources 12 and 13). The sampling rate is 200 Hz

## Discussion

We have presented a method to generate synthetic data that characterise the visual experience of a bird in flight. To our knowledge, this is the first time that such a detailed description of the complete visual field of a large bird in flight has been generated. A similar approach was carried out for a single lovebird in Eckmeier et al. (2013), albeit at a much smaller scale and without focusing on its reproducibility to other species or individuals.

We have used three sample flights to illustrate the method and carry out behavioural analyses. Although simple, these analyses already show the potential of using our method to investigate the role of vision in bird flight. Comparable studies would be exceedingly challenging if video data from head-mounted cameras was used, given the attendant limitations on payload, resolution, field of view, and motion blur. They would also be much more limited if relying on point estimates of the bird’s gaze direction in relation to prominent visual features in the environment, rather than considering the animal’s full visual field. Additionally, our method allows us to inspect counterfactual scenarios, which we have demonstrated by comparing the rendered views from the visual coordinate system and the trajectory coordinate system. Again, this would be difficult or impossible to do using any of the other reviewed approaches.

### Key Features of the Rendering Method

We have described how to model the lab environment using a hybrid approach, which combines basic geometric primitives defined using the motion capture data with dense 3D maps of features with more complex geometry. In this way, we avoid the accumulated drift and noise typical of large 3D maps, make the most of the accurate motion capture data, and reduce the modelling effort for the most intricate shapes. To facilitate the integration of the dense meshes within the basic geometric model of the lab, we transform them to the motion capture coordinate system at the point of acquisition. We have demonstrated the applicability of this hybrid approach in the pursuit flight, for the simple example of reconstructing a curtain that was hung to act as an obstacle to the bird. However, the method would be especially relevant for modelling natural-looking environments with more complicated features, such as small trees. Figure 9 illustrates this idea and shows the 3D mesh of a set of trees that we placed around the lab and captured using *SemanticPaint*, in this case using a Kinect v1 as the mapping sensor.

### Behavioural Analysis of the Pursuit Flight

During the pursuit flight, we found that the target is held within the area of the bird’s binocular overlap for most of the flight. Modelling a counterfactual gaze strategy, in which the virtual camera’s principal axis is aligned with the bird’s head velocity vector, corroborates that the bird actively directs its gaze to keep the target in this region of the visual field. Further inspecting the evolution of the target’s position in the visual field through the flight, we find that the bird fixates the target at $\pm 10^\circ$ longitude from the estimated gaze direction, and only centres it towards the terminal interception phase.

In common with most other raptors, Harris’ hawks have two areas of acute vision per retina: one projecting frontally and the other laterally (Mitkus et al., 2018; Potier et al., 2016; Inzunza et al., 1991). Where these four foveal regions (two in each retina) project on the visual field of Harris’ hawks has not been determined experimentally. For most diurnal raptors the frontal-facing foveae are estimated to project between $9^\circ$ and $16^\circ$ longitude from the forward direction of the head, and the lateral-facing fovea somewhere above $30^\circ$ longitude (Wallman & Pettigrew, 1985; Frost et al., 1990; Kane & Zamani, 2014; Tucker, 2000). It is unclear whether the frontal-facing foveae of Harris’ hawks usually project to a single point in their visual fields. For example, in Anna’s hummingbirds it has been shown that the *area temporalis*, a high resolution area in their visual fields which faces frontally, does not project to a single point, even when their eyes are fully converged (Tyrrell et al., 2018). Binocular convergence of the frontal-facing foveae does seem possible in raptors, but was rarely observed during head-restrained experiments with a little eagle. Its primary gaze position was with its frontal-facing foveae at around $13^\circ$ longitude from the head sagittal plane (Wallman & Pettigrew, 1985).

Whilst it would be premature to draw any firm conclusions from data for a single flight of a single bird, we hypothesise that these locations at $\pm 10^\circ$ longitude at which the target seems to be fixated in the pursuit flight may correspond to the projections of the frontal-facing foveae of the bird’s left and right eyes. This being so, our results could indicate that the bird tracks the target with one or other of its frontal foveae throughout the flight, before centering it in the visual field prior to interception.

### Behavioural Analysis of the Obstacle Avoidance Flights

In both obstacle avoidance flights we found that the nearside edge of the obstacles was aligned with the longitudinal centre of the visual field for substantial portions of the flights in which the obstacles were visible. Again this was not observed in the counterfactual gaze strategy that we considered, in which the virtual camera’s principal axis was aligned with the bird’s head velocity. This is in accordance with similar findings in lovebirds (Kress et al., 2015), bees (Ravi et al., 2022) and humans (Raudies et al., 2012; Rothkopf & Ballard, 2009), all of which seem to fixate on the edges of objects that can be perceived as obstacles, and on the centre of objects perceived as goals. Moreover, in the flight corresponding to the second leg of the trial, the bird seemed to align its visual field first with the edge of the landing perch and then with its centre before landing (see Online Resources 12 and 13, around frames 2029 and 2184 as numbered in the video). This is also in line with previous reports in lovebirds (Kress et al., 2015) and may reflect a strategy based on aiming at intermediate goals. It is important to note that the alignment with the perch centre is inevitable as we approach the set of frames that we used to calibrate the bird’s gaze direction (Fig. 8). However, the coincidence of the edge of the obstacles with the centre of the visual coordinate system provides strong internal support for the reliability of the visual coordinate system calibration.

An alternative explanation for the bird’s observed gaze behaviour around the obstacles is that the bird aligns with the direction where it expects the landing perch to appear. This could be the case for example in the flight executed in leg 1 of the trial, in which the edge of the landing perch is very close to the nearside edge of the obstacles (Fig. 8). Would the bird fixate on the edge of the obstacle if the perch was partially visible from the start? This could be tested directly, varying the position of the obstacles relative to the landing perch. The results obtained using the bird’s gaze strategy in this scenario could be compared to the results obtained assuming alternative gaze strategies, such as continuous fixation on the obstacles’ edge or continuous fixation on the landing perch’s edge or centre. In any case, as with the pursuit flight, these hypotheses on the animal’s behaviour can only be confirmed or rejected by analysing the complete set of flights across different individuals.

### Limitations and Future Work

We aimed to develop a reconstruction method that would enable the collection of large amounts of data from many individuals. Some key steps that we have taken towards this goal include our non-invasive tracking of head pose using marker-based motion capture, our programmatic definition of geometric primitives based on motion capture markers placed on objects in the environment, and our automated integration of the dense 3D maps on the motion capture coordinate system. The key bottleneck in our current pipeline is the need to calibrate the bird’s visual coordinate system against the coordinate system of the headpack. Currently we achieve this using two different methods, one for each manoeuvre, but both sharing common assumptions.

An alternative approach that would likely improve the accuracy of the estimated visual coordinate system would involve integrating calibrated stereo cameras with the motion capture system. Using video-based motion tracking tools (Nath et al., 2019; Pereira et al., 2022) on data collected during a calibration trial, the 3D coordinates of easily identifiable features on the bird (such as its bill tip or its eyes) could be determined in the headpack coordinate system. In this way, the bird’s head pose could be directly estimated relative to the headpack coordinate system; a similar approach was demonstrated in the recent work by Naik et al. (2020). Even if this method still assumes the bird’s eyes are fixed relative to the head, it would provide an improved estimate of the true forward direction of the bird’s head, and of the midpoint between the bird’s eyes (the ideal origin of the visual field sphere, see Martin , 2007). It would also allow us to estimate the bird’s stereo baseline, and thus define the animal’s view as a binocular system in Blender. More importantly, it provides a common calibration method independent of the recorded manoeuvre, and if properly automated, it would allow us to scale up the analyses to much larger datasets. This will be a key focus of our future work. Other improvements to further streamline the method could be automating the correction of the dense 3D maps (potentially using the object’s motion capture markers as fiducials) or improving the ArUco plate, for better floor alignment.

Tracking the eye movements of birds in flight would also be of interest to define a more accurate visual coordinate system (Holmgren et al., 2021). It would allow us to explore changes in the bird’s visual field configuration in flight (Tyrrell et al., 2018), precisely inspect how the birds make use of the high-resolution areas in their visual field (Potier et al., 2016), or investigate whether the hawks can track targets simultaneously and independently with each eye, as it has been shown for grackles (Yorzinski, 2021). However, eye-tracking in flight seems currently very challenging; in birds it has only been used in relatively large species doing terrestrial tasks (Yorzinski et al., 2013; Yorzinski & Platt, 2014; Yorzinski et al., 2015; Yorzinski, 2019).

Alternative definitions of the virtual camera or adaptations of its synthetic outputs can also provide a better approximation of the bird’s visual system. For example, the resolution of the camera could be defined non-uniformly across the visual field to more closely represent the bird’s higher visual acuity at the foveae. This would reduce rendering time and may also provide insight into what information is required by the bird at high resolution to solve the task (Matthis et al., 2018). If the RGB renderings were to be used as stimuli for birds in VR experiments (Eckmeier et al., 2013), it may be relevant to adapt them attending to the animals’ spectral sensitivity (Tedore & Johnsen, 2017; Lind et al., 2013). Considering an event-based virtual camera could also be relevant to analyse the bird’s behaviour (Mueggler et al., 2017; Rebecq et al., 2018). These bio-inspired cameras output a spike (‘event’) when pixel-level brightness changes are detected, which makes them fast and very interesting for low-latency control in flying robots (Gallego et al., 2022). Analysing the bird’s gaze strategy using the event stream of its visual experience may facilitate potential applications to autonomous drones (Rodriguez-Gomez et al., 2022; Zhu et al., 2021).

**Fig. 12:**
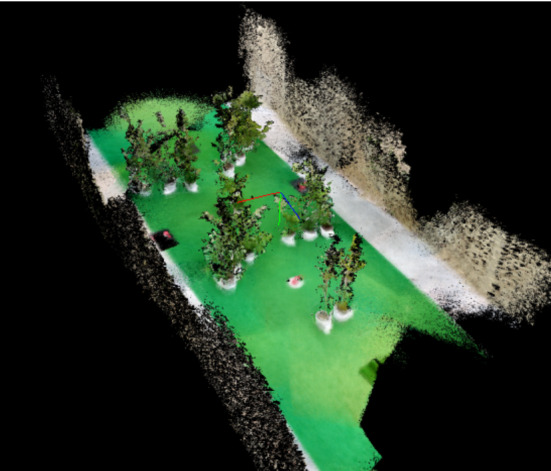
3D model of a forest environment in the lab. We used a mix of 20 small laurel and bay trees of under 2 m height to recreate a forest environment in the motion capture lab. The 3D model was captured using *SemanticPaint* and a Kinect v1 as a mapping sensor. Note that in this case the world coordinate system of the captured map is not aligned with that of the motion capture coordinate system

The method described here is primarily designed around a motion capture system, but a relevant development going forward would be to translate this method to the field. Bio-loggers combining GPS and IMU units could be used to track the bird’s head position and orientation (Kano et al., 2018), although differential GPS or a similar technology would be required to obtain sufficient location accuracy (Keshavarzi et al., 2021; Sachs, 2016). To reconstruct the natural environment, laser scanners, structure-from-motion or photogrammetry techniques may be used (Tuia et al., 2022; Stürzl et al., 2015; Stuerzl et al., 2016; Schulte et al., 2019). Consumer-level handheld mapping devices like the one used here could be useful, as they have been shown to reconstruct forest environments with reasonably accurate results (Tatsumi et al., 2022; Gollob et al., 2021). On the other hand, there are still interesting research questions to address in the lab. For example, we could examine whether the gaze strategy of the bird is affected by the familiarity or the novelty of the elements in the scene, and consider the role of top-down or bottom-up attention mechanisms in their control of gaze. Similar questions have been explored in stationary owls, albeit in a lab environment not representative of their habitat (Lev-Ari & Gutfreund, 2018; Hazan et al., 2015). A set of lab experiments with a ‘simulated forest’ like the one shown in Fig. 9 could provide this approximation, and be very useful for comparison with experiments in the field. However, to minimise the risk of occluded motion capture markers in the simulated forest would likely require reducing the volume of interest and carefully considering the cameras’ arrangement, potentially with many cameras placed right overhead.

**Table 1:** Description of supplementary videos

Trial		Filename	Description (output, coordinate system tracked, projection)
Pursuit		ESM_1.avi	RGB, visual coordinate system, orthographic
		ESM_2.avi	RGB, visual coordinate system, equirectangular
		ESM_3.avi	Depth, visual coordinate system, orthographic
		ESM_4.avi	Semantic, visual coordinate system, orthographic
		ESM_5.avi	Optic flow, visual coordinate system, orthographic
		ESM_6.avi	RGB, trajectory coordinate system, orthographic
		ESM_7.avi	RGB, trajectory coordinate system, equirectangular
Obstacle avoidance	Leg 1	ESM_8.avi	RGB, visual coordinate system, orthographic
		ESM_9.avi	RGB, visual coordinate system, equirectangular
		ESM_10.avi	RGB, trajectory coordinate system, orthographic
		ESM_11.avi	RGB, trajectory coordinate system, equirectangular
	Leg 2	ESM_12.avi	RGB, visual coordinate system, orthographic
		ESM_13.avi	RGB, visual coordinate system, equirectangular
		ESM_14.avi	RGB, trajectory coordinate system, orthographic
		ESM_15.avi	RGB, trajectory coordinate system, equirectangular

We provide supplementary videos for the rendered output per trial. The description field in the table specifies the output data represented in the video, the coordinate system the virtual camera is tracking in the video and the projection used

These suggestions show how there is still much to learn about how animals interact with their environment, and innovative methods such as the one we describe open a wide range of possibilities for behavioural analysis. Human active vision has already inspired robotic applications (Seara & Schmidt, 2004; Seara et al., 2002, 2001), as active observers have been shown to solve basic vision problems more efficiently than passive ones (Aloimonos et al., 1988). Similarly, understanding the role of active vision in bird flight may reveal efficient processing strategies that could be translated to autonomous systems. Additionally, large datasets collected with this or similar methods could support data-driven models of behaviour and offer new insights into the bird’s gaze strategy in flight; similar approaches already exist that make use of human motion capture data to generate active-sensing behaviours in synthetic humanoids (Merel et al., 2020). In conclusion, we see many exciting opportunities in the future for mutual collaboration between the animal behaviour and computer vision communities.

**Supplementary information**. We provide the rendered outputs as video supplementary material. For the pursuit flight, we include videos for the RGB, semantic, depth and optic flow synthetic data generated. For the obstacle avoidance flights, we include RGB videos. All videos are reproduced at 20Hz, (1/10 of the real speed), except the optic flow video which is reproduced at 5 Hz (1/40 of the real speed). The frame numbering shown in the videos follows the motion capture data system’s numbering. A description of each of the video files is presented in Table 1.

**Fig. 26:**
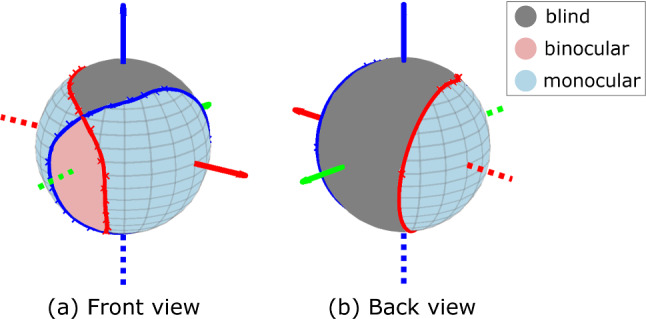
Retinal margins of Harris’ hawks eyes. The retinal margins for the left (blue) and right (red) eyes are shown on the unit sphere in the visual coordinate system, from the front (**a**) and the back (**b**). The data points for the retinal margins from Potier et al. (2016) are represented by crosses. The blind (grey), binocular (pink) and monocular (blue) areas are shown. The parallels on the sphere are plotted every $9^\circ$ in latitude and the meridians every $18^\circ$ in longitude (Color figure online)

The RGB outputs are presented using two projections: equirectangular, in which the geometry appears distorted, but which shows the complete field of view of the bird; and orthographic, in which the distortion is reduced but doesn’t include the most peripheral regions of the bird’s visual field. The rest of the rendered outputs are only represented in orthographic projection. Note that for the orthographic case, the point of view is as if looking frontally to the bird’s visual field sphere (see Fig. 26). In all videos, a red contour around the figure indicates that the head transform for that frame was interpolated. The retinal margins and the areas of the bird’s visual field (blind, monocular and binocular) are overlaid on the rendered output. The virtual camera is also defined with uniform resolution over its visual field, but note that this is not the case for the birds (see Sect. 2.2.1).

In the optic flow video, the colormap represents for each frame the instantaneous angular speed per pixel, in degrees per second. The colorbar is in logarithmic scale and capped at $10^\circ$ s$^{-1}$ in the lower bound and $1000^\circ$ s$^{-1}$ in the upper bound. The vector field results from the transformation of the output data from pixel space to the surface of the unit sphere. Further details on the computation of the videos are included in Appendix E.

**Table 2:** Birds used in the experiments

Bird	Sex	Age (years)	Mass (kg)	Manoeuvre
Toothless	Male	1.4	0.7	Pursuit
Drogon	Male	3.5	0.6	Obstacle avoidance

The name, sex, age and mass of the birds at the time of their experiments is shown, along with the recorded manoeuvre

### Supplementary Information

Below is the link to the electronic supplementary material.

Supplementary file 1 (avi 40245 KB)

Supplementary file 2 (avi 66694 KB)

Supplementary file 3 (avi 35934 KB)

Supplementary file 4 (avi 33642 KB)

Supplementary file 5 (avi 46728 KB)

Supplementary file 6 (avi 34267 KB)

Supplementary file 7 (avi 44961 KB)

Supplementary file 8 (avi 29914 KB)

Supplementary file 9 (avi 68364 KB)

Supplementary file 10 (avi 29980 KB)

Supplementary file 11 (avi 68962 KB)

Supplementary file 12 (avi 25802 KB)

Supplementary file 13 (avi 59390 KB)

Supplementary file 14 (avi 25740 KB)

Supplementary file 15 (avi 59693 KB)

## Figures and Tables

**Fig. 13:**
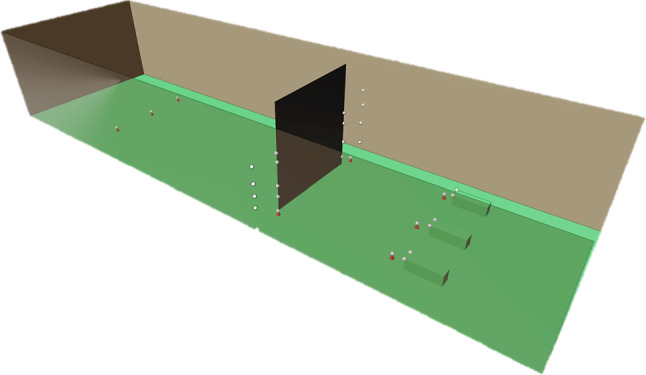
Reference motion capture markers used in the pursuit trial. The median position of the reference markers in the pursuit trial is shown in a basic geometric model of the lab environment. The sidewalls are shown as partially transparent and the curtain is modelled as a plane, for clarity. The spheres representing the markers’ positions are 5 times the markers’ real size. Note that the walls’ location was estimated from the motion capture cameras’ positions and orientations, rather than from the markers placed at the curtain gaps

**Fig. 14:**
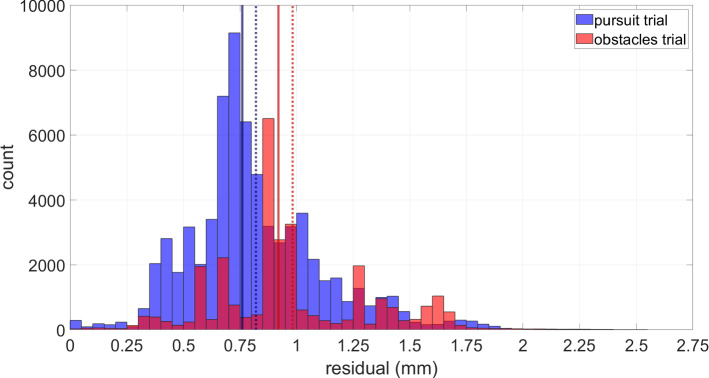
Histogram of 3D point residuals. The residuals are computed by the motion capture system as part of the reconstruction step, in which the markers’ 3D position is determined. The results for the rendered pursuit trial (blue) and obstacle avoidance trial (red) are shown. The obstacle avoidance trial consists of the two obstacle avoidance flights analysed in the main text. The mean (dashed line) and median (continuous line) values for both distributions are under 1 mm. Note that all markers and recorded frames per trial are considered. The bins’ width is 0.05 mm

**Fig. 15:**
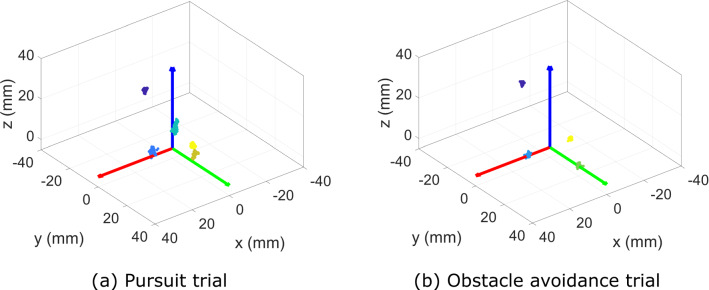
Labelled markers per trial in a local coordinate system. The labelled markers for each of the pursuit (**a**) and the obstacle avoidance trials (**b**) are shown in a coordinate system that moves with the headpack. In (**a**) the visual coordinate system is used and in (**b**) the headpack coordinate system is shown; both correspond to the template used for procrustes labelling in each trial. We considered all rendered frames in which the headpack transform was not interpolated. Note that we used different headpack designs for the two trials. The obstacle avoidance trial consists of the two obstacle avoidance flights analysed in the main text

**Fig. 16:**
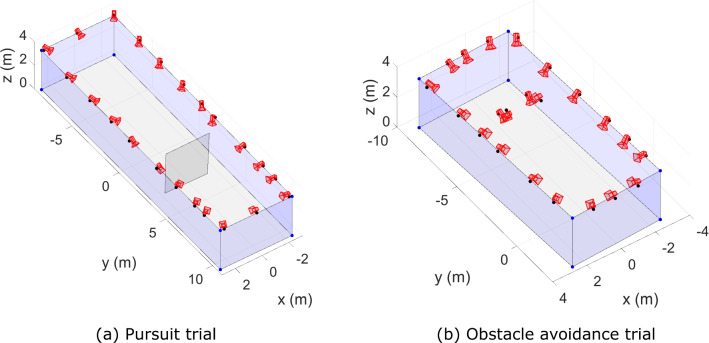
Estimated location of the walls. We estimated the location of the walls from the motion capture cameras’ positions and orientations for the pursuit flight (**a**) and the obstacle avoidance flights (**b**). Cameras are shown in red. From the dimensions of the cameras and their mounts, we estimated points in the scaffolding (black markers). We then used the scaffolding points to the define the walls’ planes (blue transparent planes). The corners of the volume are highlighted with blue markers. A plane representing the curtain’s location is shown for reference in (**a**), computed from the markers attached to it

**Fig. 17:**
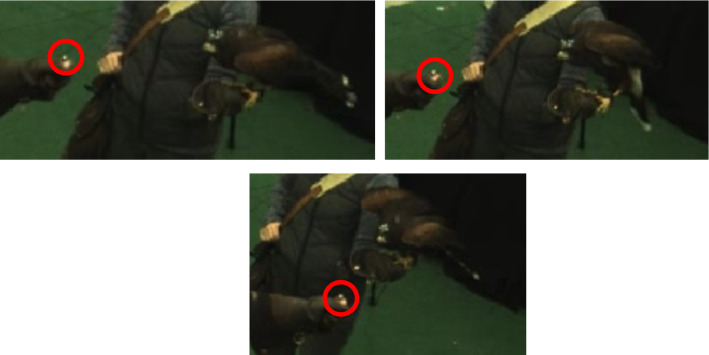
Examples of the bird’s behaviour in the fixation phase of the gaze calibration trial. For certain periods during the gaze calibration trial, the bird appeared to focus on the food reward presented to it, with a characteristic behaviour of lowering its head and preparing its wings for a downstroke. The marker on the food reward is highlighted with a red circle

**Fig. 18:**
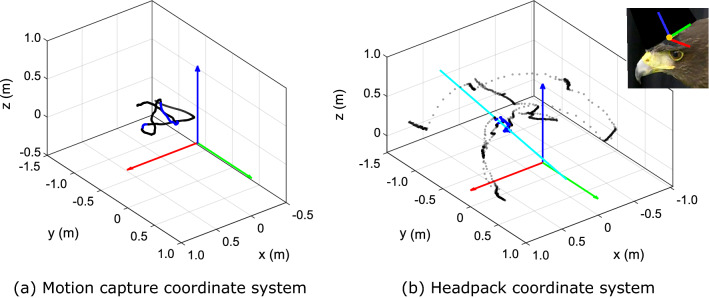
Estimation of gaze direction for the pursuit flight. The trajectory of the marker attached to the food reward in the gaze calibration trial is represented in two coordinate systems: one that translates with the headpack and is parallel to the motion capture coordinate system (**a**), and one fixed to the headpack (**b**). The estimated gaze direction $\vec {v}_{gaze}$ is the orthogonal regression line (cyan) to the samples in the fixation phase (blue) in the headpack coordinate system (**b**). The samples outside this phase are shown for reference (black, semi-transparent). The food reward is presented to the bird at a distance between 0.5 and 1.5 m. Note how the position of the food marker in the fixation phase in (**b**) falls within a small region, in contrast to the curved trajectory it follows in (**a**), suggesting the bird is actively positioning the food reward on a small area of its visual field during that phase. Inset in (**b**) shows the approximate orientation of the headpack coordinate system relative to the bird’s head

**Fig. 19:**
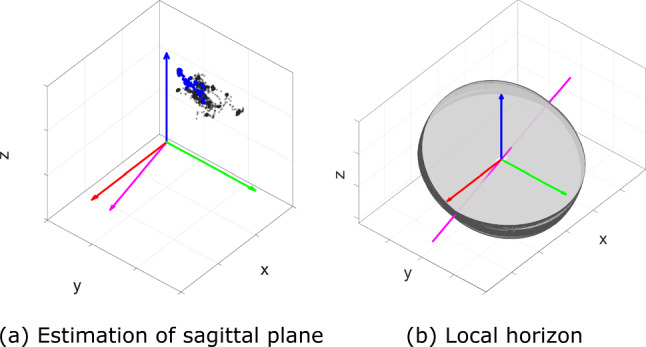
Estimation of the normal to the sagittal plane for the pursuit flight We estimated the normal to the sagittal plane $\vec {n}_{sagittal}$ (magenta), assuming the bird keeps its eyes level during the fixation phase of the gaze calibration trial. In (**a**), dots represent the direction of the world’s z-axis in the headpack coordinate system, during the fixation phase (blue markers) and outside the fixation phase (black, semi-transparent). Samples outside the fixation phase show larger variation in roll (i.e. rotation around the y-axis). In (**b**), the local horizon (i.e. the plane perpendicular to the world’s z-axis in the headpack coordinate system) is represented for every frame in the fixation phase (gray circular planes). The direction of the $\vec {n}_{sagittal}$ vector (magenta) is the best-fit line that is perpendicular to the previously estimated $\vec {v}_{gaze}$ direction and is contained in all these planes, in least-squares sense

**Fig. 20:**
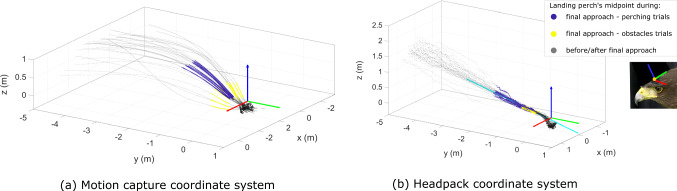
Estimation of the gaze direction for the obstacle avoidance flights. The gaze direction $\vec {v}_{gaze}$ is estimated over 8 trials with obstacles in place (obstacle trials) and 7 trials without them (perching trials). Note that each trial consists of two flights, which correspond to the two legs of the trial (see Fig. 3b). The landing perch’s midpoint (grey and coloured dots) is represented from 5 m away until landing, in two coordinate systems: one that translates with the headpack and is parallel to the motion capture coordinate system (**a**), and one fixed to the headpack (**b**). The estimated gaze direction $\vec {v}_{gaze}$ is the orthogonal regression line (cyan) to the samples in the final approach phase (plotted blue for perching trials, and yellow for obstacle trials) in the headpack coordinate system (**b**). Note the straightness of the perch’s midpoint trajectories in the headpack coordinate system in (**b**), and compare this to the curvature of the lines in (**a**), which confirms unequivocally that the head pose was being stabilised in relation to the perch. Inset in (b) shows the approximate orientation of the headpack coordinate system relative to the bird’s head

**Fig. 21:**
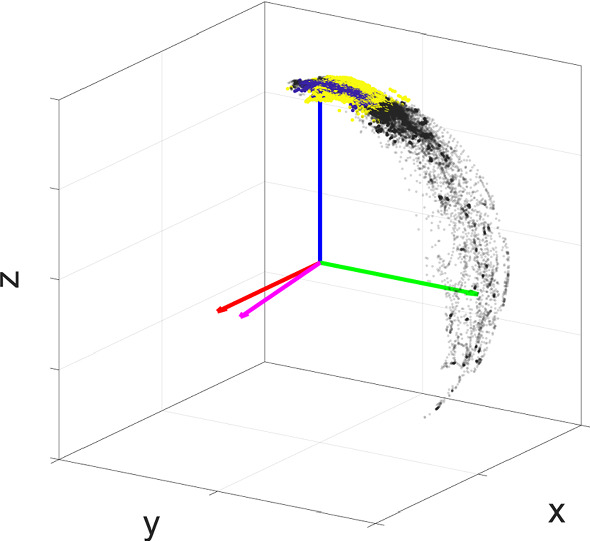
Estimation of the normal to the sagittal plane for the obstacle avoidance flights. We estimated the normal to the sagittal plane $\vec {n}_{sagittal}$ (magenta) assuming the bird keeps its eyes level during the mid-flight phase. The dots represent the direction of the world’s z-axis in the headpack coordinate system during the mid-flight phase, for perching (blue) and obstacle trials (yellow). Note that each trial consists of two flights, which correspond to the two legs of the trial (see Fig. 3b). Samples outside of the mid-flight phase (black, transparent), show qualitatively larger variation in pitch (i.e., rotation around the x-axis) and roll (i.e., rotation around the y-axis)

**Fig. 22:**
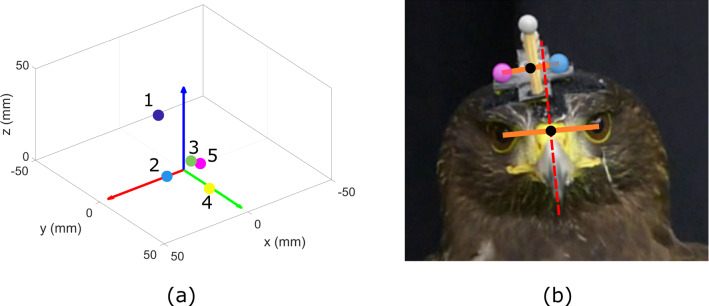
Distance between selected origin and midpoint between the eyes for the pursuit flight, in the direction perpendicular to the sagittal plane. Figure (**a**) shows the disposition of the markers in the headpack coordinate system. The origin (which is the same for the headpack, visual and trajectory coordinate systems) lies at the midpoint between markers 2 (blue) and 5 (magenta). A front view of the bird wearing the headpack is shown in (**b**), with the orange segments highlighting the line between markers 2 (blue) and 5 (magenta), and the approximate line between the eyes. The midpoints of these segments are shown as black dots. The trace of the sagittal plane (red dashed line) contains the midpoint between the eyes, and we estimate it based on the symmetry of the bill. We can see that the distance between the sagittal plane and the selected origin is smaller than the distance between the origin and marker 5 (10 mm)

**Fig. 23:**
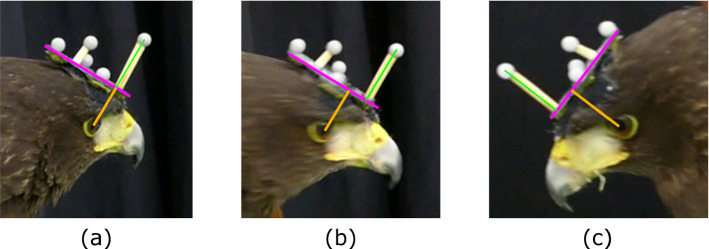
Distance between selected origin and midpoint between the eyes for the pursuit flight, in the direction perpendicular to the headpack’s plate. We selected three frames in which the headpack baseplate was almost perpendicular to the camera plane. We then estimated on each of them the real length of the yellow segment (the approximate distance from the headpack’s baseplate to the bird’s eye), based on the known length of the green segment (the height of marker 1 above the baseplate). We obtained a mean value of 20.1 mm

**Fig. 24:**
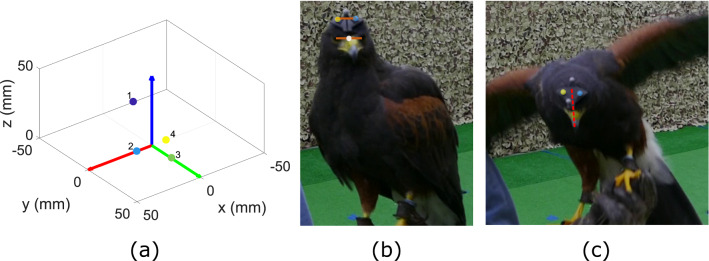
Distance between selected origin and midpoint between the eyes for the obstacle avoidance flights, in the direction perpendicular to the sagittal plane. Figure (**a**) shows the disposition of the markers in the headpack coordinate system. The origin (which is the same for the headpack, visual and trajectory coordinate systems) lies approximately between markers 2 (blue) and 4 (yellow). A front view of the bird wearing the headpack is shown in (**b**), with the line between markers 2 (blue) and 4 (yellow) and the approximate line between the eyes. The midpoint of the line between the eyes (white dot) falls within the segment connecting markers 2 and 4. We therefore estimate that the distance between the midpoint between the eyes and the origin is at most, the maximum distance between the origin and one of the highlighted markers (12 mm). In (**c**), the red dashed line drawn following the bill’s symmetry shows the approximate location of the sagittal plane, which contains the midpoint between the eyes

**Fig. 25:**
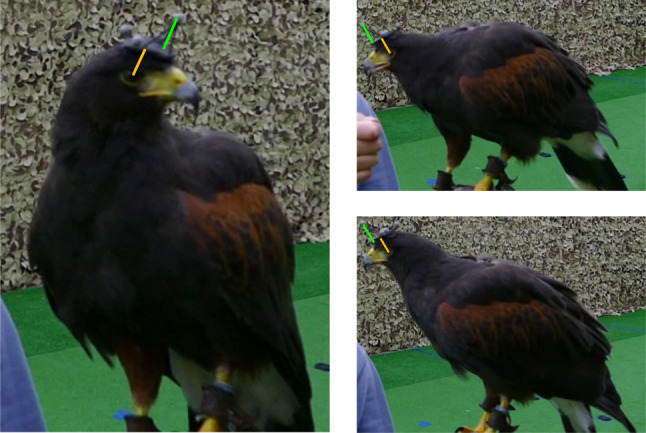
Distance between selected origin and midpoint between the eyes for the obstacle avoidance flights, in the direction perpendicular to the headpack’s plate. We selected three frames in which the bird’s sagittal plane was almost parallel to the camera plane. We then estimated on each of them the real length of the yellow segment (the approximate distance from the headpack’s baseplate to the bird’s eye), based on the known length of the green segment (the height of marker 1 above the baseplate). We obtained a mean value of 17.3 mm

**Fig. 27:**
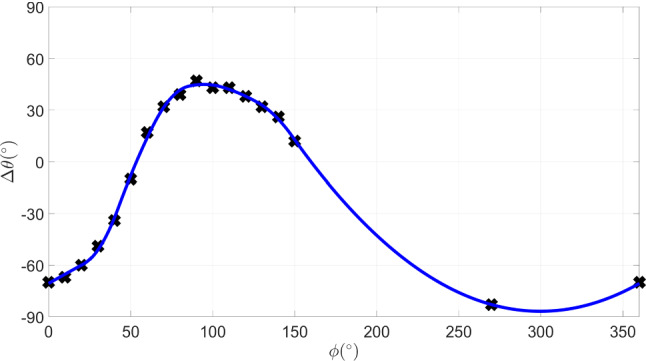
Overlap between retinal margins of Harris’ hawks’ eyes. We digitized the overlap between the retinal margins of the bird’s eyes ($\Delta \theta$) at several angles measured from the top of its head ($\phi$). We fitted the data (black crosses) to a smoothing spline (blue dots) with periodic boundary conditions (RMSE = 1.6$^\circ$)

**Fig. 28:**
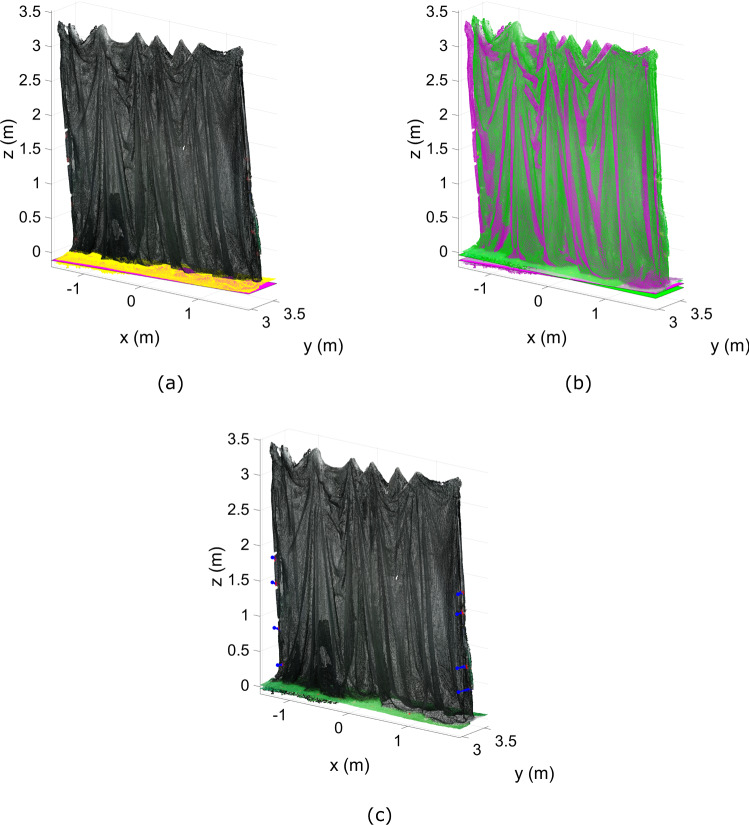
Correction of the captured curtain mesh. We fitted a plane to the floor of the mesh; (**a**) shows the fitted plane (magenta) and the inlier points (yellow). We rotated the mesh so that its fitted floor plane was parallel to the floor plane of the motion capture coordinate system; (**b**) shows the original mesh and the fitted floor plane (magenta), and the rotated mesh and floor plane (green). We then translated the rotated mesh so that its floor plane contained the origin of the motion capture system (not shown). (**c**) shows the median position of the markers fixed to the curtain during the pursuit trial (blue dots) and the segments (blue lines) connecting them to the nearest vertices from the red subset of the mesh (highlighted in red) (Color figure online)

**Fig. 29:**
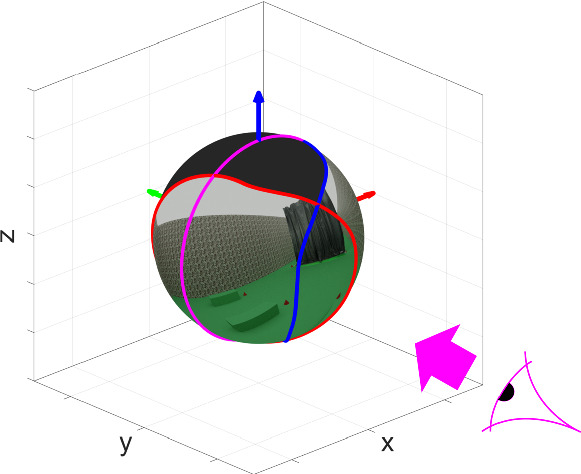
Orthographic projection of the rendered output. The point of view of the rendered videos in orthographic projection is represented schematically. The rendered output (here, the RGB data) is represented in the visual field unit sphere of the bird. The retinal margins for the left (blue) and right (red) eyes are represented, as well as the blind area (black). The direction of the orthographic projection is represented with a magenta arrow and an eye icon. Note that when projecting the data as indicated by the arrow, the most peripheral parts of the field of view of the bird (beyond the magenta contour) are not included (Color figure online)

## Appendix A: Motion Capture Experiments

### A.1: Motion Capture System

The motion capture lab is equipped with 22 infrared motion capture cameras (Vantage V16, Vicon Motion Systems Ltd, Oxford, UK; sampling rate 200 Hz) and four video cameras (Vue, Vicon Motion Systems Ltd, Oxford, UK; sampling rate 100 Hz, only used for reference). The cameras were mounted on a fixed scaffold from which camouflage netting was hung, to provide a natural-looking background texture and prevent the birds from perching on the lower parts of the scaffolding. The floor was carpeted with green astroturf.

### A.2: Bird Flight Experiments

The flights presented in this paper were recorded with two different captive-bred adult Harris’ hawks: Drogon (0.6 kg) flew the obstacle avoidance flights, and Toothless (0.7 kg) the pursuit flight. Further details on the birds are provided in Table 2. The birds were housed separately in external aviaries, fully roofed with open fronts, receiving full natural light and water *ad libitum*. The hawks were exercised in free flight at least 5 times a week and baths were provided daily.

The pursuit flight is part of a dataset we recorded with four different birds, in which we collected between 3 and 4 flights per bird per day, during four weeks between December 2018 and January 2019, for a total of 251 trials. The number of flights per bird and day in the pursuit dataset was limited by the training approach: an extra food reward was required to get the bird off the target and back to the falconer’s fist, which increased the food intake per trial. This reduced the amount of flights we could record while keeping the animals motivated to fly. Resetting the setup between trials also took more time than when recording perching or obstacle avoidance trials.

The obstacle avoidance flights are part of a dataset we recorded with four different birds (Drogon, Toothless, Charmander and Ruby), in which we collected 16 flights per bird per day for a total of two weeks in November 2020. Both periods include the training weeks for the birds.

### A.3: Placement of Markers

To track the birds’ head movements, we used rigid supports of retroreflective markers for the birds to wear as a ‘headpack’. In the pursuit flight, we used a headpack of 5 retroreflective markers, made of wooden thin rods glued to a plastic cross-shaped plate. In the obstacle avoidance flights, we specially designed 3D-printed rigid supports for 4 retroreflective markers. Both designs were under 5 g, which is less than 1% of the birds’ mass (see Table 2), so we don’t expect the headpacks to have an impact on the animals’ natural behaviour.

We used 4–5 markers even if only three markers are required to extract the pose of a rigid body (in our case, the headpack attached to the bird’s head). One the one hand, additional markers allow us to derive the pack’s pose even if at some frames some markers are not reconstructed correctly. On the other hand, too many markers constrained to a small volume (such as the top of the bird’s head) are more likely to occlude each other in several motion capture camera views, which may lead to an incorrect determination of the markers’ 3D position. We therefore considered 4–5 markers to be a good trade-off between these two constraints.

The headpack was attached to the head of the bird by the falconer immediately before recording its set of flights, and removed at the end of the set. To ensure that the headpack did not move relative to the bird’s head we recorded videos of the bird on the falconer’s fist wearing the headpacks. For the flights analysed here, we did not detect visually any movement between the headpack and the head. The head movements when the bird is stationary are generally of larger amplitude than those observed in flight, so we consider these videos are a good indication that there was no significant relative motion between the bird’s head and the headpack in flight.

In the pursuit flight, we fixed markers of 6.4–10 mm diameter to the pulleys’ centres, to the top front vertices of the starting boxes, to the edges of the curtain (which were reinforced with a wooden rod) and to the camouflage netting at the curtain gap. Figure 10 shows the median position of the reference markers used in the pursuit flight; note that the walls’ positions were estimated with the motion capture cameras’ position and orientation, rather than with the markers. To track the target’s motion, we glued three markers (6.4 mm diameter) to its canvas cover. In the obstacle avoidance flight, we placed 14 mm diameter markers to the perches’ edges and at the centre of the obstacles’ tops.

### A.4: Experimental Procedure

In the pursuit trial, a linear motor pulled the target on a line passing around a series of pulleys. The trajectory of the target was randomised by selecting for each trial a set of pulleys, from one of three starting positions to one of three end positions, via one of the two gaps around the curtain (Fig. 3a). The curtain was made of two layers of flame-proofed cotton canvas of size 6.0 m $\times \, 3.5$ m, and hang from a steel cable installed transversally across the motion capture room. The line was a 3 mm thick parachute-style cord, in green to minimise contrast with the astroturf carpet, and the average speed of the target was 5.6 m s$^{-1}$. To keep the target in tension, a long piece of line was attached to its back end and rolled around the first pulley of the target’s trajectory. We also randomised the takeoff position of the bird and placed dummy lines along the alternative paths, to minimise the risk of the bird predicting the target’s path. The bird began flying freely from its falconer’s fist as soon as the target appeared from inside the starting box in which it was hidden. Before recording the pursuit trial, we recorded a ‘gaze calibration trial’ to estimate the bird’s gaze direction within the headpack coordinate system. In it the falconer held the bird on their fist whilst we displayed a piece of food with a marker attached at a range of 0.5–1.5 m.

The obstacle avoidance flights are part of a set in which we recorded the bird’s head movements as it flew from the starting perch to the end perch, and back, with and without obstacles in place. For each trial we recorded two flights, which corresponded to the trajectories back and forth between the perches. Two falconers stood at either end of the room, to handle the bird and provide the food reward. The lateral position of the perches was randomised for every trial between three stations each. These were centred on the longitudinal axis of the room and distributed with 1 m spacing. Each obstacle was made up of two white expanded polystyrene cylinders of 1 m height, stacked on top of each other and bound together with white duct tape. The four obstacles were pushed together so that there were minimal gaps between them. For the trials with obstacles in place, we also randomised the side of the end perch from which the falconer would call the bird. We used the complete set of flights from the same bird on the same day to calibrate the bird’s gaze direction within the headpack coordinate system (see Sect. 2.2).

We calibrated the motion capture system using an active wand and following the manufacturer guidelines. In the obstacle avoidance set of trials, we calibrated before recording a set of trials with the same bird and headpack placement. In the pursuit dataset, we calibrated the system without the curtain in place, and before recording all trials per day. Since we recorded less trials in the pursuit dataset, we don’t expect the calibration to deteriorate significantly (the flight presented here is recorded 1.5 hours after calibrating)

## Appendix B: Motion Capture Data Postprocessing

### B.1: Markers’ Reconstruction and Labelling

We used the commercial software Vicon Nexus 2.8.0, from the motion capture system manufacture, to extract the unlabelled 3D coordinates of the retroreflective markers per frame. This process is called marker reconstruction. The system provides a residual value for each reconstructed marker in 3D space to evaluate the measurement accuracy (Motion Lab Systems, 2021). Figure 11 shows the histogram of residuals for the trials rendered in this paper. For the pursuit trial, the mean residual is 0.82 mm, the standard deviation is $\sigma =0.29$ mm, and the median is 0.76 mm, for $n = 69537$ samples. For the obstacle avoidance trial, which consists of the two obstacle avoidance flights considered, the mean residual is 0.98 mm, the standard deviation is $\sigma =0.34$ mm, and the median 0.92 mm, for $n = 29403$ samples. All markers and recorded frames per trial are included in the computation of the histograms (i.e., frames recorded before takeoff and after landing are also considered).

Nexus is designed for collection and annotation of 3D motion capture data, with a particular focus on human motion. We found that our experiments pushed the system to its limits in terms of tracking and labelling performance, and therefore considered alternative options to process our data. For a detailed description of the typical challenges of using marker-based motion capture on birds the reader is referred to Naik (2021).

For the pursuit flight, we labelled the individual headpack markers per frame using the Nexus software’s semi-automatic labelling tool. For the target, it was difficult to separate the individual identities of the markers with manual labelling, as the target twisted and turned during the trial. Instead we assigned a consistent set of three labels to the target markers, that allowed us to separate them easily from the rest. We exported the manual labels to MATLAB, and wrote custom scripts to label the remaining markers.

For the obstacle avoidance dataset, we wrote custom MATLAB scripts to separate headpack and object markers, and label them. To label the headpack markers, we used the following approach:

- First, we labelled the frames with at least 4 markers by iteratively solving the orthogonal procrustes problem with the headpack template; we also determined the headpack’s transform (position and orientation) at those frames.
- Second, we interpolated the headpack’s transform at the remaining frames; and
- Third, we iteratively labelled the markers in the remaining frames, by assigning them the label of the closest marker of the corresponding interpolated headpack.

We iterated over the third step, keeping a constant minimum distance for accepting a label but updating the interpolated transform data for the headpack with the newly labelled frames every time. We stopped when there were no new frames labelled between two passes. To label the markers in the objects, we used predefined bounding boxes.

To assess the quality of our labelling of the headpack markers, we computed the maximum distance between a labelled marker and its corresponding position in the headpack template, in a local coordinate system. Results are shown in Fig. 12 and Table 3. Although the ideal distance deviation without measurement error would of course be zero, the actual distance deviation is in all cases smaller than the diameter of the headpack markers, which implies that the markers are correctly labelled, albeit that their precise positioning is subject to measurement error. In line with our observations during manual labelling, the pursuit trial appears to have more jitter in the markers’ positions (even though the residuals obtained from the system are not much larger, see Fig. 11). For the set of trials with the same bird and headpack placement as in the selected obstacle avoidance trial (which we used to estimate the visual coordinate system), the maximum distance registered across all trials was 3.7 mm.

**Table 3:** Maximum distance $d_{max}$ between labelled markers and corresponding markers in headpack template

Label	$d_{max}$ (mm)	
	Pursuit trial	Obstacle avoidance trial
1	1.8	1.6
2	3.1	2.0
3	4.6	2.8
4	2.2	1.6
5	1.6	N.A.

We considered all rendered frames in which the headpack transform was not interpolated. Note that we used different headpack designs for the two trials, and therefore the same label does not correspond to the same marker. The obstacle avoidance trial consists of the two obstacle avoidance flights analysed in the main text

### B.2: Headpack Transform Per Frame

To compute the headpack’s transform per frame, we solved the procrustes problem on the labelled data. In the pursuit flight, we used a template computed from the gaze calibration data. In the obstacle avoidance flights, we used the theoretical headpack design as the template.

For the frames where there were not enough headpack markers reconstructed, or where these could not be reliably labelled, we interpolated the headpack’s transform. In the pursuit flight, we used a smoothing cubic spline to interpolate the headpack’s translation. In the obstacle avoidance flights, we used a weighted variation that takes into account the number of markers detected. In both cases we interpolated the headpack’s rotation using the SLERP algorithm for quaternions, which assumes constant angular velocity (Shoemake, 1985). We assumed the short path between quaternions and constant pose in the extrapolation regions.

During manual labelling of the pursuit flight we found that some of the markers’ reconstructions were jittery and noisy for certain sections, so we computed the procrustes transform on a subset of frames we determined as most reliable, and interpolated the translation and rotation of the headpack for the rest of the frames. The subset of frames considered most reliable is the set of frames with 3 or more markers reconstructed, excluding those frames in which the procrustes error is above a certain threshold, the markers are close to collinear, or the most prominent frontal marker is missing. We then applied a low-pass filter to the resulting head rotations, as a moving average filter of window size equal to 2 frames, using the SLERP algorithm:

2 $$\begin{aligned} q_{f}(t) = slerp(q_{f}(t-1),q(t),p), \end{aligned}$$

where $q_{f}(t)$ denotes the filtered quaternion at frame *t*, *q*(*t*) denotes the quaternion data point, *slerp* denotes the implementation of the SLERP algorithm in MATLAB, and *p* is the interpolation parameter, which we set at 0.5. For the first frame $q_{f}(0)=q(0)$. We applied the same filter when computing the head rotations for the trajectory coordinate system.

### B.3: Target Transform Per Frame

For the pursuit flight, we modelled the target as a cylinder, with the three markers attached to it defining a right-angled triangle. We defined a coordinate system linked to the cylinder, whose origin is at the cylinder’s centre, whose y-axis is parallel to the longitudinal axis of the cylinder (pointing inwards), and whose x and z-axis are two perpendicular radii of the cylinder.

We computed the translation of the cylinder as the trajectory of the circumcentre of the triangle. We estimated it using a cubic smoothing spline, that takes into account the number of markers reconstructed.

We define the rotation of the cylinder’s coordinate system as follows: its y-axis is always parallel to the origin’s velocity vector $\vec {v}_{origin}$, and its x-axis is always parallel to the floor plane of the lab. This mimics the actual motion of the target, which was in tension between two lines, one pulled by the linear motor and one looped around the initial pulley.

We consider the interception frame to be the instant at which the distance from the bird to the target first reaches a local minimum in the final approach phase.

### B.4: Walls, Floor and Ceiling Geometry

The motion capture cameras were mounted on a fixed scaffold from which camouflage netting was hung. We estimated its location using the position and orientation of the motion capture cameras. These were computed during the calibration. We used the cameras’ positions and orientations to estimate points on the scaffolding, taking into account the dimensions of the cameras and their mounts. We then fitted the estimated scaffolding points to a line for each wall section. This provided an estimate of the scaffolding rungs holding the netting and the cameras. We defined each wall as the plane perpendicular to the floor that contained the corresponding scaffolding rung, from which the camouflage netting was hung.

**Table 4:** Results of the gaze direction fitting, for the pursuit and obstacle avoidance flights

Trial	Bird	$\vec {v}_{gaze}$	$A_{gaze}$ (mm)	*d* (mm)	RMSE (mm)	*n*
Pursuit	Toothless	$$\begin{bmatrix} 0.16,&-0.94,&0.32 \end{bmatrix}$$	$$\begin{bmatrix} 103.7,&-572.3,&251.5 \end{bmatrix}$$	54.3	47.5	462
Obstacles	Drogon	$$\begin{bmatrix} 0.13,&-0.94,&0.31 \end{bmatrix}$$	$$\begin{bmatrix} 131.4,&-969.9,&247.0 \end{bmatrix}$$	68.4	65.6	1274

The estimated gaze direction $\vec {v}_{gaze}$ and the point in the fitted line $A_{gaze}$ are both expressed in the headpack coordinate system. The distance between the fitted line and the origin of the headpack coordinate system is *d*. RMSE stands for root mean square error, where the error is the distance between the sample points and the fitted line. The number of samples per fit is denoted by *n*. For the pursuit flight, it corresponds to the number of frames in the fixation phase in the gaze calibration trial. For the obstacle avoidance flights, it corresponds to the number of frames in the final approach phase, across all the flights recorded with that bird and headpack placement

Figure 13 shows the estimated walls and the motion cameras for the pursuit and the obstacle avoidance flights. The mean angle at the walls’ corners was $90^\circ$, with standard deviation $\sigma =0.2$ for the pursuit flight, and $\sigma =0.5$ for the obstacle avoidance flights. We set the ceiling at the mean height of the estimated scaffolding points: 3.33 m for the pursuit flight and 3.25 m for the obstacle avoidance flights.

## Appendix C: Visual Coordinate System Calibration

To define the visual coordinate system relative to a headpack coordinate system, we made use of three assumptions (see Sect. 2.2). We identified specific periods in the collected data in which we expect these assumptions to hold most reliably. We used the data during these periods to estimate $\vec {v}_{gaze}$, the bird’s gaze direction, and $\vec {n}_{sagittal}$, the normal to the bird’s head symmetry plane (i.e., the sagittal plane). These vectors define the basis of the visual coordinate system: the x-axis is defined parallel to $\vec {n}_{sagittal}$ (pointing to the left side of the head) and the y-axis parallel to $-\vec {v}_{gaze}$. We defined the x and y axes in this way so that the visual coordinate system is close to parallel to the motion capture coordinate system at the start of the trial; this facilitates the interpretation of the computed rotations. The following sections describe the estimation of $\vec {v}_{gaze}$ and $\vec {n}_{sagittal}$ for the pursuit and the obstacle avoidance flights.

### C.1: Estimate for the Pursuit Flight

For the pursuit flight, we estimated the visual coordinate system using data from the gaze calibration trial. During this trial, we recorded the bird’s head movements while it was held on the falconer’s fist and presented with a food reward that had a marker attached to it. In the corresponding reference video, we identified a ‘fixation phase’: a range of frames in which the bird is likely to be fixating on the food reward while holding its head level. We identified these frames by a characteristic behaviour of the bird, in which it lowers its head and prepares its wings for a downstroke; three samples of the typical posture of the bird in this phase are shown in Fig. 14. We selected two sequences of 342 and 120 frames of motion capture data within the trial in which this behaviour was most apparent.

We estimated the bird’s forward gaze direction $\vec {v}_{gaze}$ by fitting a 3D line to the food marker’s trajectory during the fixation phase, in an auxiliary coordinate system linked to the headpack (RMSE = 47.5 mm for $N=462$ samples); the resulting vector is shown in Table 4 and Fig. 15.

For the same range of frames, we estimated the orientation of the imaginary line connecting the bird’s eyes, assuming the animal keeps its head approximately level during that period. We identify this line with $\vec {n}_{sagittal}$, the normal to the bird’s head symmetry plane (i.e. the sagittal plane), which we define as positive when pointing to the left side of the bird’s head. We compute $\vec {n}_{sagittal}$ as the unit vector perpendicular to $\vec {v}_{gaze}$ that best approximates the normal to the sagittal plane in the headpack coordinate system. We do this by solving the following least-squares problem:

3 $$\begin{aligned} \theta ^*&= \arg \min _{\theta } \sum _{i=1}^{N} (\vec {z}_{world,i} \cdot \vec {n}_{sagittal}(\theta ))^2, \nonumber \\&\quad \vec {n}_{sagittal}(\theta ) = (cos\theta ) \vec {a} + (sin\theta )\vec {b}, \nonumber \\&\qquad \vec {n}_{sagittal} = \vec {n}_{sagittal}(\theta ^*), \end{aligned}$$

where $\vec {z}_{world,i}$ is the world’s z-axis in the headpack coordinate system (positive opposite to gravity), $\vec {a}$ and $\vec {b}$ are an arbitrary orthonormal basis of the plane perpendicular to $\vec {v}_{gaze}$, and $\theta$ is the angle between $\vec {n}_{sagittal}$ and $\vec {a}$. The fit over the $N=462$ fixation frames yields a root-mean square residual of 0.04; the resulting vector is shown in Table 5 and Fig. 16.

The origin of the visual coordinate system in the pursuit flight is defined as the midpoint between the two lateral markers of the headpack (see Fig. 19a). We estimated this point to be close to the midpoint between the eyes, the ideal origin of the visual coordinate system (Potier et al., 2016). From reference images of the headpack on the bird’s head, we estimate that the origin is $< 10$ mm from the midpoint between the bird’s eyes in the direction perpendicular to the sagittal plane, and $<20.1$ mm in the direction perpendicular to the headpack’s baseplate (see Appendix 1).

**Table 5:** Results of the normal to the sagittal plane fitting, for the pursuit and the obstacle avoidance trial

Trial	Bird	$\vec {n}_{sagittal}$	RMSE	*n*
Pursuit	Toothless	$$\begin{bmatrix} 0.98,&0.11,&-0.16 \end{bmatrix}$$	0.04	462
Obstacles	Drogon	$$\begin{bmatrix} 0.99,&0.14,&-0.01 \end{bmatrix}$$	0.08	5056

The estimated normal $\vec {n}_{sagittal}$ is expressed in the headpack coordinate system. RMSE stands for root mean square error, where the error is the residual of the least-squares problem solved. The number of samples per fit is denoted by *n*. For the pursuit trial, it corresponds to the number of frames in the fixation phase in the gaze calibration trial. For the obstacle avoidance trial, it corresponds to the number of frames in the final approach phase, across all the trials recorded with that bird and headpack placement

### C.2: Estimate for the Obstacle Avoidance Flights

For the obstacle avoidance flights, we estimated the visual coordinate system using data from 15 trials with the same bird and headpack placement. Note that each trial consists of two flights, which correspond to the two legs of the trial (see Fig. 3b). From those 15 trials, 8 were recorded with obstacles in place, and 7 without them. We identified two phases in all the flights within this set of trials: a final approach phase, in which we expect the bird to fixate on the centre of the perch; and a mid-flight phase, in which we expect the bird to keep its head level. We used these two phases to estimate $\vec {v}_{gaze}$ and $\vec {n}_{sagittal}$, respectively.

We estimated the bird’s forward gaze direction $\vec {v}_{gaze}$ using data from the final approach phase. This phase was defined based on the bird’s distance to the landing perch (0.5–1.0 m for the obstacle trials, 0.5–2.0 m for the no obstacle trials; note that the obstacles were placed 1.5 m ahead of the end perch, as per Fig. 3b). We fitted a 3D line to the trajectory of the midpoint of the landing perch during this phase, in a headpack coordinate system (see Fig. 17). The root-mean square error of the fit was RMSE = 65.6 mm, for N = 1274 samples; the resulting vector is shown in Table 4. Results were similar to those obtained by fitting each trial individually (see Appendix 1, Table 6).

We computed the normal to the sagittal plane $\vec {n}_{sagittal}$ following the same approach as in the pursuit flight, using the data of the mid-flight phase. We defined this phase to be when the bird was $>2$ m away from either perch and flying at a speed $>2.5$ m s$^{-1}$; for all flights these conditions defined a continuous range of frames. We solved for $\vec {n}_{sagittal}$ using the set of equations in 3, and obtained a root-mean square residual of 0.08 for $N=5056$ samples; the resulting vector is shown in Table 5 and Fig. 18.

We defined the origin of the visual coordinate system as the centroid of the headpack markers projected onto the headpack’s baseplate plane. This is different to the definition used in the pursuit flight because we used a different headpack design. From reference images, we estimate this point is within 12 mm of the midpoint between the bird’s eyes in the direction perpendicular to the sagittal plane, and $< 20$ mm in the direction perpendicular to the headpack’s baseplate (see Appendix 1).

### C.3: Distance Between the Origin and the Midpoint Between the Eyes

In the pursuit flight, the origin of the visual coordinate system is defined as the midpoint between the two lateral markers of the headpack (see Fig. 19a). In the obstacle avoidance flights, it is defined as the centroid of the headpack markers projected onto the plane defined by the headpack baseplate (see Fig. 21a; note that the headpack designs are not the same in the pursuit flight and the obstacle avoidance flights). We selected these points aiming to be close to the midpoint between the eyes. We estimated how much these points deviate from the midpoint between the eyes using snapshots of the videos of the birds wearing the headpacks, recorded before and after the trials.

The reference images used to estimate this offset for the pursuit flight are shown in Figs. 19 and 20. Figure 19a shows the selected origin lies between markers 2 and 5, and Fig. 19b shows it is closer to the sagittal plane (red dashed line) than to marker 5. We therefore estimate that the offset in the direction perpendicular to the sagittal plane is, at most, the distance between the origin and marker 5, which is 10 mm. To estimate the offset in the direction perpendicular to the headpack’s baseplate, we selected three frames in which the baseplate plane was almost perpendicular to the camera plane. We estimated for each of them the real length of the yellow segment, based on the known real length of the green segment, and obtained a mean value of 20.1 mm.

We followed the same approach for the obstacle avoidance flights; the reference images used are shown in Figs. 21 and  22. From these we estimate that the midpoint between the eyes is within 12 mm from the selected origin in the transverse direction (perpendicular to the sagittal plane), and 17.3 mm in the direction perpendicular to the headpack baseplate.

### C.4: Gaze Direction Fit

To estimate $\vec {v}_{gaze}$ in both the pursuit and the obstacle avoidance trials, we computed the orthogonal regression line to the assumed points of fixation in the headpack coordinate system. We used the singular-value decomposition approach described in Söderkvis (2021):

4 $$\begin{aligned} U S V^T&= svd(X - \overline{X}), \end{aligned}$$

5 $$\begin{aligned} \vec {v}_{gaze}&= U(:,1), \end{aligned}$$

6 $$\begin{aligned} A_{gaze}&= \overline{X} \end{aligned}$$

where *X* denotes the array of sample points (size $3\times N$, with *N* the number of sample points) and $\overline{X}$ is the mean of the sample points (size $3\times 1$). The singular-value decomposition is represented by the operator *svd*(). The matrices *U*, *S*, *V* correspond to the matrix containing the left singular vectors in columns, the diagonal matrix containing the singular values, and the matrix containing the right singular vectors in columns, respectively. The estimated gaze direction unit vector $\vec {v}_{gaze}$ is computed as the first column of *U*. $A_{gaze}$ is the centroid of the sample points, and a point in the fitted line. Table 4 shows the complete fitting results for the flights considered in the main text.

The root mean square error (RMSE) in Table 4 is computed with the error being the distance between the samples and the fitted line. We additionally computed the distance *d*, between the origin of the headpack coordinate system and the fitted line (Table 4).

For both the pursuit and the obstacle avoidance trial, the distance *d* is larger than the distance we estimated between the origin of the headpack coordinate system and the midpoint between the eyes in the previous section, Appendix 1. Therefore, we don’t expect either of these lines to closely represent a line that goes through the midpoint between the eyes. This could be due to the bird fixating on a different points than the ones we assume, due to eye movements that we are not accounting for, or due to existing movement of the headpack relative to the head. We expect this last effect to be minimal, as we didn’t find any evidence of it in the reference videos. In any case, we expect the estimated visual coordinate system to be more representative of the forward direction of the head and of the sagittal plane orientation than a coordinate system simply aligned to the headpack. The fact that our preliminary results seem in agreement with observations previously reported in the literature would support this, although it should be confirmed with more flights.

For the obstacle avoidance trial, we checked for every trial recorded with the same bird and headpack placement, whether the slope of the fitted line per trial was similar across trials. The results are shown in Table 6. We computed a deviation metric $\sigma _{\vec {v}_{gaze}}$ for the estimated unit gaze vector per trial, as the square-root of the trace of the covariance matrix:

7 $$\begin{aligned} \sigma _{\vec {v}_{gaze}} = \sqrt{\sigma _{{v}_{x,gaze}}^2 +\sigma _{{v}_{y,gaze}}^2 + \sigma _{{v}_{z,gaze}}^2} , \end{aligned}$$

obtaining $\sigma _{\vec {v}_{gaze}} = 0.0463$. The mean gaze vector was $$\overline{\vec {v}_{gaze}} = \begin{bmatrix} 0.11,&-0.95,&0.29 \end{bmatrix}$$, very similar to the gaze direction estimate considering all trials aggregated (second row in Table 4). We also computed the distance from the origin of the headpack coordinate system to the fitted line per trial, which yielded a mean value of $\overline{d}=65.56$ mm and a standard deviation of $\sigma _{d}=22.88$ mm, showing the variability in the landing perch midpoint trajectories per trial.

**Table 6:** Results of the gaze direction fitting per trial, for all trials with the same bird and headpack placement as the rendered obstacle avoidance trial

Trial	$\vec {v}_{gaze}$	$A_{gaze}$ (mm)	*d* (mm)	RMSE	*n*
Drogon perching 1	$$\begin{bmatrix} 0.10&-0.94&0.33 \end{bmatrix}$$	$$\begin{bmatrix} 136.50&-1108.30&306.00 \end{bmatrix}$$	84.78	21.94	122
Drogon perching 2	$$\begin{bmatrix} 0.13&-0.92&0.37 \end{bmatrix}$$	$$\begin{bmatrix} 112.38&-1086.61&317.02 \end{bmatrix}$$	113.15	48.07	123
Drogon perching 3	$$\begin{bmatrix} 0.17&-0.93&0.33 \end{bmatrix}$$	$$\begin{bmatrix} 212.42&-1094.46&310.33 \end{bmatrix}$$	74.83	37.44	122
Drogon perching 4	$$\begin{bmatrix} 0.12&-0.95&0.28 \end{bmatrix}$$	$$\begin{bmatrix} 122.18&-1138.82&285.44 \end{bmatrix}$$	46.50	44.70	117
Drogon perching 5	$$\begin{bmatrix} 0.13&-0.96&0.25 \end{bmatrix}$$	$$\begin{bmatrix} 196.22&-1115.80&263.51 \end{bmatrix}$$	50.17	29.78	117
Drogon perching 6	$$\begin{bmatrix} 0.12&-0.93&0.36 \end{bmatrix}$$	$$\begin{bmatrix} 149.82&-1095.98&315.56 \end{bmatrix}$$	99.12	54.63	124
Drogon perching 7	$$\begin{bmatrix} 0.09&-0.96&0.27 \end{bmatrix}$$	$$\begin{bmatrix} 172.20&-1126.35&248.55 \end{bmatrix}$$	91.52	43.80	112
Drogon obstacles 1	$$\begin{bmatrix} 0.12&-0.96&0.26 \end{bmatrix}$$	$$\begin{bmatrix} 141.08&-686.26&172.10 \end{bmatrix}$$	59.95	62.09	57
Drogon obstacles 2	$$\begin{bmatrix} 0.07&-0.95&0.29 \end{bmatrix}$$	$$\begin{bmatrix} 65.88&-707.29&154.66 \end{bmatrix}$$	62.08	50.43	54
Drogon obstacles 3	$$\begin{bmatrix} 0.09&-0.96&0.28 \end{bmatrix}$$	$$\begin{bmatrix} 19.18&-711.67&158.51 \end{bmatrix}$$	65.75	48.99	56
Drogon obstacles 4	$$\begin{bmatrix} 0.15&-0.96&0.26 \end{bmatrix}$$	$$\begin{bmatrix} 117.33&-699.39&160.86 \end{bmatrix}$$	29.74	61.56	58
Drogon obstacles 5	$$\begin{bmatrix} 0.11&-0.96&0.27 \end{bmatrix}$$	$$\begin{bmatrix} 85.59&-709.25&155.57 \end{bmatrix}$$	44.30	15.51	53
Drogon obstacles 6	$$\begin{bmatrix} 0.09&-0.96&0.27 \end{bmatrix}$$	$$\begin{bmatrix} 32.18&-710.18&156.70 \end{bmatrix}$$	57.27	24.38	55
Drogon obstacles 7	$$\begin{bmatrix} 0.12&-0.96&0.27 \end{bmatrix}$$	$$\begin{bmatrix} 109.94&-697.48&153.10 \end{bmatrix}$$	50.87	73.59	55
Drogon obstacles 8	$$\begin{bmatrix} 0.12&-0.95&0.28 \end{bmatrix}$$	$$\begin{bmatrix} 83.00&-707.49&154.79 \end{bmatrix}$$	53.41	20.28	49

The estimated gaze direction $\vec {v}_{gaze}$ and the point in the fitted line $A_{gaze}$ are both expressed in the headpack coordinate system. The distance between the fitted line and the origin of the headpack coordinate system is *d*. RMSE stands for root mean square error, where the error is the distance between the sample points and the fitted line. The number of samples *n* corresponds to the number of frames in the final approach phase, for each trial

#### C.4: Retinal Margins

The retinal margins define the set of directions in the visual field that project on the right or left retina. We represented the retinal margins of Harris’ hawks’ eyes in the estimated visual coordinate system, using data from Potier et al. (2016) (Fig. 23).

The authors in Potier et al. (2016) measured the visual field experimentally, aligning the bird’s sagittal plane with a visual perimeter and using an ophthalmoscopic reflex technique. They determined the degree of overlap ($\Delta \theta >0$) or divergence ($\Delta \theta <0$) between the retinal margins of the bird’s eyes, at several angles measured from the top of its head $\phi$. We digitized the data from the paper (figures 5C and 6 from Potier et al. , 2016) and fitted a smoothing spline with periodic boundary conditions (8 polynomial pieces of order 2) to the relation $\Delta \theta$ vs $\phi$ (RMSE = 1.6$^\circ$, Fig. 24). Note that the data point at $\phi =360^\circ$ is just a duplicate of the data point at $\phi =0^\circ$. From the interpolated overlap $\Delta \theta$ we derived the retinal margins for each eye, $\theta _{left}$ and $\theta _{right}$, assuming symmetry with respect to the sagittal plane. We also assumed our estimated gaze direction corresponds to the $\phi$ angle where the binocular overlap is widest ($\phi = 90^\circ$) (in line with the point raised in the supplementary figure S1 from Potier et al. , 2016).

### C.5 Trajectory coordinate system

We define the trajectory coordinate system as a coordinate system with its y-axis tangent to the forward direction of the trajectory and its x-axis parallel to the horizontal:

8 $$\begin{aligned} \vec {y}_{T}&= - \frac{\vec {v}}{||\vec {v}||}, \end{aligned}$$

9 $$\begin{aligned} \vec {x}_{T}&= \frac{\vec {y}_{T} \times \vec {z}_{world}}{||\vec {y}_{T} \times \vec {z}_{world}||}, \end{aligned}$$

10 $$\begin{aligned} \vec {z}_{T}&= \vec {x}_{T} \times \vec {y}_{T}, \end{aligned}$$

where $\vec {x}_{T},\vec {y}_{T},\vec {z}_{T}$ represent the versors of the trajectory coordinate system, $\vec {v}$ represents the velocity vector of the head, and $\vec {z}_{world}$ represents the world z-axis. We computed the velocity vector of the head $\vec {v}$ with a central differences scheme on the head interpolated trajectory using the *gradient* function in MATLAB. In the pursuit trial, we filtered the rotations of the trajectory coordinate system using the same approach applied to the visual coordinate system.

## Appendix D: Model of the Lab Environment

### D.1: Geometry of Pursuit Flight

The pulleys used to guide the target’s trajectory were diabolos of 6.5 cm radius and 15 cm length, glued to a metallic base of 1 cm width. We reduced their geometry in Blender to cones of 6.5 cm radius at the base, and height determined by the corresponding reference markers placed at the diabolos’ axes. Some pulleys had additional markers placed on the contour but these were not used to model their geometry.

We modelled the starting boxes as cuboids of 1 m length, with their height and orientation defined by the reference markers placed at their top front vertices.

We modelled the target as a cylinder of 15 cm length and 2.54 cm diameter, based on reference images of the markers’ location on the target and on the average distances measured between the markers over the whole trial (see Table 7). In determining the target’s width, we took into account the diameter of the markers attached to it (6.4 mm).

We rendered one trial in which we added a texture to the wall of the motion capture room, that mimicked the texture of the camouflage netting. We did this by tiling a picture of the netting with appropriate scaling. The results are presented in the Online Resources 1 and 2.

**Table 7:** Mean distances between the markers fixed to the target

	Mean value (mm)
Length	91.2
Width	31.8
Diagonal	97.4

Computed from applying *k-means* clustering to the distances between markers labelled as belonging to the target. Length, width and diagonal refer to the dimensions of the cylinder with which we approximate the target’s shape

### D.2: Geometry of Obstacle Avoidance Flights

We defined the geometry and locations of the perches and obstacles based on the positions of their corresponding markers (placed at the perches’ edges and at the centre of the obstacles’ tops).

We reduced the perches to their top rungs and modelled them as cylinders of radius 4 cm (based on measurements of the actual perches). From reference images we estimated that a line between the centres of the markers on the perch’s edges would be approximately tangent to the top rung cylinder.

We modelled the obstacles as vertical cylinders of 0.3 m diameter. We estimated the offset between the larger markers’ centre and the obstacles’ tops by computing the mean deviation from 2 m height for these markers. We took this offset into account (14 mm, including the markers’ base) when defining the obstacles’ dimensions in Blender.

### D.3: Dense 3D Map of the Curtain

**Table 8:** Distances between the motion capture markers fixed to the curtain and the subset of red vertices in the curtain’s mesh

	Pursuit trial		Mapping trial	
	Original mesh	Transformed mesh	Original mesh	Transformed mesh
Mean (cm)	9.7	9.3	9.1	8.9
Minimum (cm)	5.2	5.7	5.7	5.9
Maximum (cm)	16.4	16.6	13.0	13.3

The values shown represent the maximum, minimum and mean distance (in cm) between the median position of the curtain markers and the 20 nearest neighbours in the red vertices subset of the curtain’s mesh

The 3D mapping was carried out after collecting all bird trials of the day. To evaluate the error in the captured mesh, we recorded a motion capture trial just before carrying out the 3D mapping, that registered the static motion capture markers in the scene and on the ArUco calibration plate. We call this the mapping trial. We noticed the markers fixed to the curtain weren’t clearly identifiable in the captured meshes, so we placed red tape around them to signal their approximate region prior to mapping. These pieces of tape were also in place during the bird trials.

We started the mapping procedure by setting up a local wireless network between the motion capture computer, the augmented reality smartphone and the laptop running *SemanticPaint*, making use of its streaming functionality. We then started the *SemanticPaint* application on the laptop and connected the smartphone’s augmented reality app (*Tango Streamer*) to it. Once the connection was verified, the ArUco calibration plate was brought into camera view to compute the required coordinate system transformation. After visually confirming the computed coordinate system for the mesh was correct, the mapping was carried out.

To obtain the 3D map for the curtain, we cropped it from a partial mesh of the lab using MeshLab (Cignoni et al., 2008). We cleaned the resulting mesh by removing duplicate vertices and isolated pieces. We noticed that the floor plane in the captured mesh was slightly deviated from the motion capture coordinate system’s floor so we corrected the mesh in MATLAB to match them.

To do this we first fitted a plane to the floor of the mesh with a reference normal equal to [0, 0, 1], using *pcfitplane* in MATLAB. This is an implementation of the M-estimator sample consensus algorithm (MSAC), a variant of RANSAC. The plane was computed with a 99% confidence of finding the maximum number of inliers, with a maximum distance between an inlier point and the plane of 10 cm, and a maximum angular distance between the normal vector of the fitted plane and the reference orientation [0, 0, 1] of $2.5^\circ$. The resulting angle between the fitted plane and the reference orientation was $2.4^\circ$ and the mean distance between inlier points and the fitted plane was 3.8 cm.

We rotated and translated the input mesh so that the fitted floor plane contained the origin of the motion capture system, and its normal was parallel to [0, 0, 1]. This implied the following transforms:

11 $$\begin{aligned} R&= \begin{pmatrix} 1.00 &{} 0.00 &{} -0.04 \\ 0.00 &{} 1.00 &{} 0.005 \\ 0.04 &{} -0.005 &{} 1.00 \end{pmatrix}, \end{aligned}$$

12 $$\begin{aligned} \vec {t}&= \begin{bmatrix} 0.00,&0.00,&3.53 \end{bmatrix} \end{aligned}$$

where *R* denotes the rotation matrix applied and $\vec {t}$ the translation vector, in centimetres.

We computed the deviation from the reference motion capture markers as follows. For the markers placed on the curtain edges, we computed their reference position as the median of its coordinates across all frames, for the pursuit trial and for the mapping trial. From all the points in the mesh, we selected a subset of 400 vertices, whose colour was closest to red (i.e., the vertices whose RGB vector had lowest sum-of-square error from the red vector, [255, 0, 0]). For each reference marker, we computed the mean distance to the 20 nearest vertices from the red subset (see Fig. 25). The mean, maximum and minimum distances for the original and the transformed mesh are shown in Table 8.

## Appendix E: Supplementary Videos

Figure 26 shows a representation of the orthographic projection used in the rendered videos. With an orthographic projection the distortion is less than if we use an equirectangular projection, but the most peripheral parts of the bird’s field of view are not included. Both projections are used to represent the RGB data in the supplementary videos. A description of the supplementary videos is summarised in Table 1.

The data was rendered using two GPUs model NVIDIA 3090RTX. Rendering the two obstacle avoidance flights (814 frames) took approximately 48 minutes, including all possible synthetic outputs exported as multilayer exr files and a jpeg file per frame for preview. Rendering the same outputs for the pursuit flight (516 frames), in a scene with the dense map for the curtain but no texture in the walls, took approximately 70 minutes.

For the pursuit trial, we exported the RGB channel in PNG format, and the rest of channels as part of a multilayer OpenEXR file. In the frame numbering shown in the video, the split between the two phases of the trial (before and after the curtain) is at frame 1132, and the interception frame corresponds to 1374.

For the obstacle avoidance trial, we exported all rendered passes as a mulitlayer OpenEXR file. For the RGB video, we used the *tonemap* function in MATLAB to read the HDR images. The analysis presented in the paper excludes the first and last 20 frames of the videos for each leg of the trial. In the frame numbering shown in the video, the landing perch becomes fully visible at frame 958 in leg 1, and frame 1997 in leg 2 of the trial. The observed saccades in the second leg of the trial occur at approximately frame 2029 and frame 2184. These are more evident in the orthographic projection.

We used the OpenEXR bindings available at https://github.com/skycaptain/openexr-matlab to read the OpenEXR files in MATLAB.
